# How conspiracy theories are defined: A systematic qualitative review of academic definitions

**DOI:** 10.12688/openreseurope.23324.1

**Published:** 2026-05-08

**Authors:** Türkay Salim Nefes, Javier Martínez Escobar, Borja González-Albo

**Affiliations:** 1 The Institute of Public Goods and Policies (IPP), Consejo Superior de Investigaciones Cientificas, Madrid, Community of Madrid, 28037, Spain; 2 Information Management Uni, Consejo Superior de Investigaciones Cientificas, Madrid, Community of Madrid, 28037, Spain

**Keywords:** Conspiracy theories; citation analysis; definitions; conceptual variation; qualitative analysis, systematic review.

## Abstract

**Background:**

Although conspiracy theories are studied across multiple disciplines, their definition remains unsettled. Despite a substantial body of empirical research, definitions have not been systematically analysed, leaving it unclear how conceptual variation shapes research, methods, and findings.

**Methods:**

Drawing on an original dataset of peer-reviewed publications, this study applies inductive qualitative content analysis to identify recurring dimensions along which academic definitions vary, including ontological assumptions, evaluative orientations, epistemic characterisations, constitutive features and scope. It further introduces a genealogical mapping of citation relationships to assess whether these dimensions structure more frequently reused definitions.

**Results:**

The content analysis shows that while many definitions converge in framing conspiracy theories as explanatory accounts involving secret coordination, they differ in how they embed evaluative and epistemic judgments. Genealogical mapping demonstrates that conceptual variation persists even among central formulations and is reproduced through citation practices.

**Conclusions:**

Definitional diversity in conspiracy theory research is maintained through citations. Making definitional choices explicit and treating definitions as analytical tools rather than background assumptions can improve conceptual clarity, comparability, and cumulative research.

## Plain Language summary

Researchers do not all agree on what a “conspiracy theory” is. Studies use different definitions, but these differences are rarely examined systematically. Yet the way a concept is defined affects how research findings are interpreted and compared.

In this study, we reviewed peer-reviewed publications that explicitly define the term “conspiracy theory.” We examined each definition to identify recurring patterns. We looked at what conspiracy theories are described as, whether definitions include value judgments, how they treat knowledge and evidence, which features are considered essential, and how broadly the concept is applied.

We found that most definitions describe conspiracy theories as explanations involving secret coordination by multiple actors. However, they differ in important ways. Some definitions include negative judgments about truth or rationality, while others remain neutral. These differences also appear among the most frequently cited definitions and are repeated over time through citations.

Overall, there is no single shared academic definition of conspiracy theory. Instead, different approaches coexist. Making these differences explicit can improve clarity, transparency, and comparability in future research and in policy discussions that rely on academic concepts.

## Introduction

Recent scholarship notes that the academic literature lacks a standard definition of conspiracy theories despite increased empirical attention ( Nera and Schöpfer, 2023; Uscinski and Enders, 2023a). This lack of consensus has consequences beyond academia. For example, the European Commission defines conspiracy theories as beliefs involving secret manipulation by powerful actors with negative intent, embedding an assumption of inherent malevolence. Such framings exclude contexts in which conspiratorial narratives are interpreted as protective: In Turkey, the ‘deep state’ has often been portrayed as a covert security mechanism aimed at preserving political order not solely as a malign force ( Gürpınar 2019). The contrasting meanings attached to conspiracy theories show that the definitions shape which narratives are recognised as conspiratorial and how their social and political significance is interpreted. This motivates a closer examination of how they are defined.

Although definitional variations are acknowledged in research, they have not been examined systematically. Studies employ diverse definitions, ranging from neutral descriptive formulations to normative ones. Definitional diversity tends to be treated as a background condition rather than as an object of analysis. Thus, without treating definitions as data, it remains unclear whether conceptual variation reflects substantive analytical differences or the uneven inheritance of earlier formulations. Addressing these gaps, this study analyses how conspiracy theories are defined by drawing on peer-reviewed articles. In so doing, it applies inductive qualitative content analysis to identify recurring dimensions. It also introduces a genealogical mapping of reference relationships to examine whether these dimensions also structure recurrent reference points. Combining qualitative analysis with genealogical mapping enables a comprehensive examination of definitional content, making it possible to assess whether conceptual diversity diminishes or persists at the core of the field. By treating definitions as objects of analysis, the study contributes to conceptual clarity and provides a foundation for more reflexive and comparable research on conspiracy theories. To address these gaps, this systematic qualitative review is guided by two research questions:

(1) How are conspiracy theories explicitly defined in peer-reviewed academic literature?
(2) Along which recurring conceptual dimensions does definitional variation occur, and how are these patterns reproduced through citation practices?

The article proceeds in four steps. First, it situates the study in the scholarship and highlights the lack of systematic analysis of definitions. Second, it describes the construction of the dataset and the inductive qualitative content analysis used to identify definitional dimensions, together with the genealogical mapping of citation relationships. Third, it presents the findings, detailing how definitions vary along ontological, evaluative, epistemic, structural, and scope-related dimensions. Finally, it discusses the implications of these patterns for conceptual clarity and outlines potential directions for future studies.

## Defining conspiracy theories: Academic research and conceptual gaps

Research on conspiracy theories has expanded rapidly over the past two decades ( Rao and Greve 2024). Reviews document extensive work on the antecedents, correlates, and consequences of conspiracy beliefs, particularly in psychology, where scholars examined cognitive and affective processes such as uncertainty, threat perception, and distrust ( Douglas et al. 2019; Douglas and Sutton 2023). Other contributions situate conspiracy theories in relation to social and political topics such as democratic governance, epistemic authority, political communication, and cultural meaning-making, drawing on philosophy, political science, sociology, and communication studies ( Douglas et al. 2019). In these strands, conspiracy theories are treated as significant phenomena, linked to political mobilisation, institutional and interpersonal distrust, and misinformation dynamics. This literature generated substantial empirical findings and theoretical insights into why conspiracy theories emerge and how they operate in social life.

Despite this growth, definitions of conspiracy theories are often weakly specified or inconsistently applied. Mahl, Schäfer, and Zeng (2023) show that many studies rely on implicit or inherited understandings of conspiracy theories, leading to conceptual ambiguity. They show that definitions are frequently taken for granted, borrowed without reflection, or omitted altogether, even as the concept is operationalised in measurement and analysis. In parallel, Uscinski and Enders (2023b) argue that the absence of clear and consistently applied definitions allows conspiracy theory to function as a flexible and often subjective label rather than a stable analytical category, enabling evaluative judgments to enter implicitly through definitional practice. Moreover, conceptual ambiguity also arises from the frequent conflation of conspiracy theories with adjacent constructs such as conspiracy mentality or suspicion. Recent empirical work distinguishes between these related but analytically distinct phenomena, showing that dispositional conspiracy mentality and belief in specific conspiracy theories do not necessarily overlap and may have different attitudinal and behavioural correlates ( Nefes and Herreros 2025). Recognising such distinctions further underscores the importance of examining how conspiracy theory itself is defined in academic research.

When definitions are explicitly made, they vary considerably. Studies posit formulations, ranging from neutral explanatory accounts involving secret coordination to normative definitions that portray conspiracy theories as epistemically deficient or socially harmful ( Douglas et al. 2019). Philosophical scholarship has engaged most directly with such definitional and epistemic questions, particularly through debates over whether conspiracy theories are intrinsically suspect or should instead be assessed on a case-by-case basis ( Clarke 2002; Coady 2006; Dentith 2016; Keeley 1999). However, this work remains largely conceptual. Valuable contributions proposed, refined, or criticised definitions in relation to broader epistemic commitments. Consequently, definitional plurality is acknowledged but typically illustrated through a small number of canonical examples rather than systematically. Overall, the scholarship illustrates that definitional work is ubiquitous and weakly documented. What is missing is a systematic map of this definitional landscape—one that captures how conspiracy theories are conceptualised and how specific formulations are reproduced over time.

Disagreements over how conspiracy theories should be evaluated are closely tied to how they are defined, and these disagreements can be approached from a sociological perspective concerned with the construction of concepts. Particularist approaches argue that conspiracy theories should be assessed on a case-by-case basis and resist treating them as a unified category, typically favouring broad and neutral definitions centred on secret coordination ( Coady 2006). Generalist approaches, by contrast, maintain that conspiracy theories can be evaluated as a class and therefore rely on more restrictive definitions that foreground shared epistemic or normative deficiencies ( Harris 2022). From a sociological standpoint, these positions reflect competing ways of constructing the object of inquiry. Definitions do not simply describe conspiracy theories; they help constitute them as a category of knowledge. As Thomas and Thomas (1928) posited, when people define situations as real, those definitions have real consequences. Building on this insight, the present study shifts attention from how conspiracy theories ought to be evaluated to how they are actually defined. Treating definitions as socially situated practices provides a basis for examining their epistemic and practical consequences and understanding how conceptual choices shape the future trajectory of conspiracy theory research as well as policies.

## Methods

Data were collected by the three authors using a systematic bibliographic search in three major databases: Web of Science (WoS), Scopus, and OpenAlex. Databases were last searched in February 2025. The search strategy targeted publications defining or conceptualising conspiracy theories using two term sets: ( *conspira* + *theory* + (defin OR concept)); ( *conspiracism* + (defin OR concept)). Searches were limited to peer-reviewed articles, review articles, and notes published in English. Using multiple databases reduces disciplinary and publication bias and increases the likelihood of capturing influential contributions. Because database search options differ, proximity operators were applied in WoS and Scopus, whereas OpenAlex required broader Boolean combinations. This resulted in a higher volume of initial records in OpenAlex, which necessitated additional manual screening to reduce informational noise. The database showing, search logic, and retrieval results are reported in Table 1 below, which underpins the subsequent screening process. All steps followed a predefined and documented review protocol.

**Table 1.** Search procedure for obtaining articles with a CT definition.

	WoS	Scopus	OpenAlex
Procedure	The databases used: Science Citation Index, Social Science Citation Index, Arts & Humanities Citation Index, and Emerging Science Citation Index. In this case, considering the following sets – a) conspira*; b) theory; c) (defin* or concept*); d) conspiracism; e) (defin* or concept*)- the search strategy establishes that terms a) and b) can be found within a maximum of three words of each other in the bibliographic fields. This set can be separated by a maximum of three words from c). Similarly, and d) and e) must be within a maximum of three words of each other. The search fields are the title, abstract, and keywords.	The same search strategy is used and with the same restrictions as for WoS.	Because the system does not allow the use of proximity operators between terms neither truncations, the single terms were considered for the topic definition, combining them with boolean operators. This results in greater informational noise in the results obtained, so it was necessary to perform an initial screening of the documents obtained. Therefore, two search strategies were used against the title and abstract fields, although they were combined into a single one for the database search: 1. (Conspiracy OR Conspiracist OR Conspiratorial) AND (theory) AND (definition OR define OR concept OR conceptualise). 2. (Conspiracism) AND (Theory) AND (definition OR define OR concept OR conceptualise).
Results	105 documents	154 documents	1638 initial documents; 159 potentially relevant

Retrieved records were consolidated and screened using eligibility criteria. All records were merged into a single database and deduplicated primarily using Digital Object Identifiers (DOIs). Where DOIs were unavailable, manual matching was conducted using title, author(s), journal, and year of publication, and the source database was retained using dummy variables. Eligibility was determined through criterion sampling ( Patton 2002). To be included in the analysis, a publication had to contain an explicit and author-endorsed definition of conspiracy theory. Publications referring to conspiracy theories only indirectly—through examples, descriptive discussions, or alternative labels such as *conspiracy narrative* or *conspiracy belief*—were excluded from the main corpus but reviewed separately to inform reflections on conceptual boundaries. Following initial screening, snowball sampling was applied by examining references cited in the included definitions to identify additional publications with definitions. This iterative process yielded an analytical corpus, the construction of which is summarised in Figure 1 below. Titles and abstracts were screened independently by two reviewers, and full-text articles were subsequently assessed for eligibility through discussion among the authors until consensus was reached.

**Figure 1.**
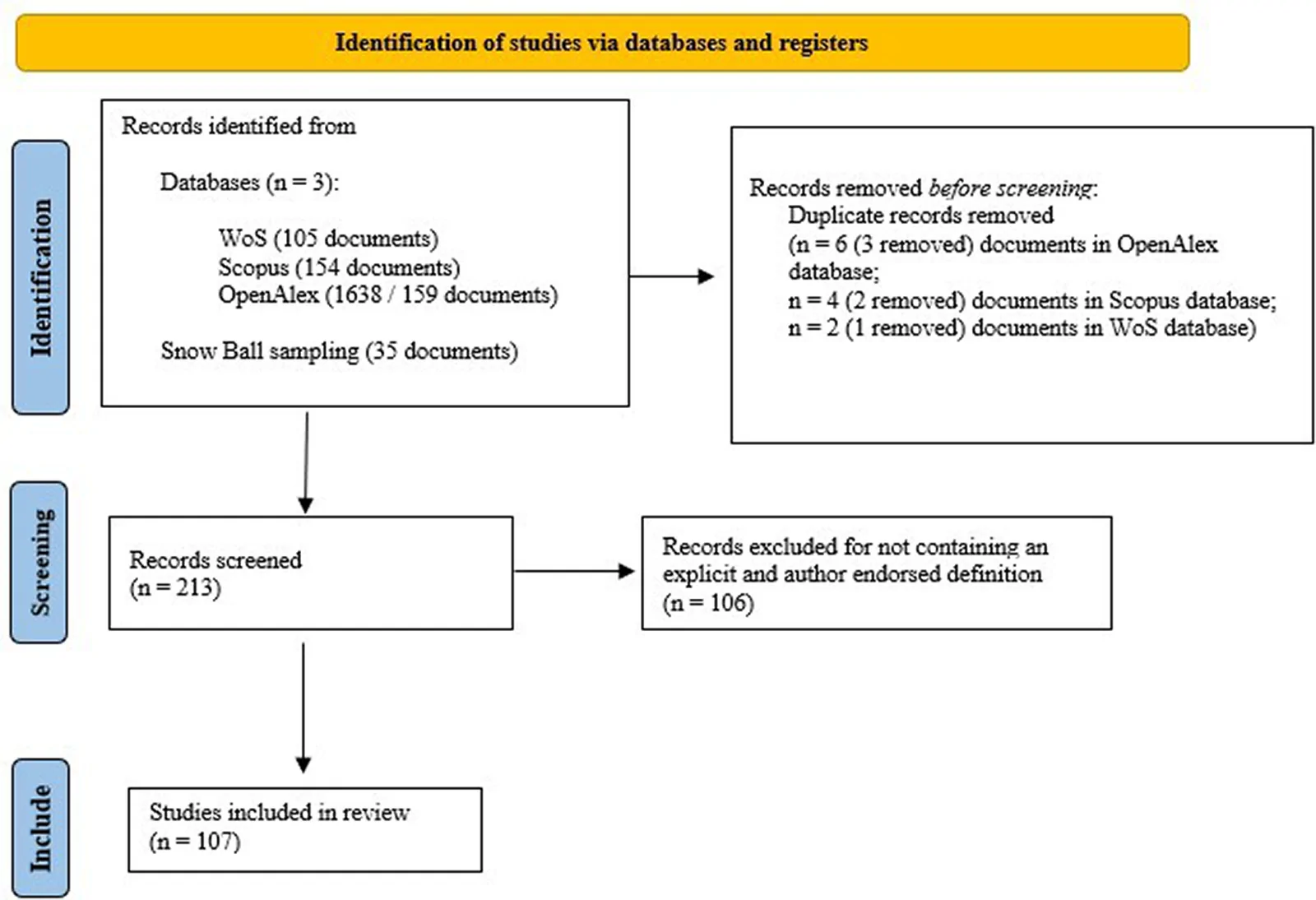
Flowchart of Identification, Screening, and Inclusion of Studies.

The final dataset ( González-Albo, Martínez Escobar and Nefes 2025) was analysed using qualitative content analysis. Data extraction and coding were conducted by the authors, with categories iteratively refined through discussion until consensus was reached. For each publication, the passage in which *conspiracy theory* was explicitly defined was treated as the unit of analysis. Identical or closely related formulations were retained as separate observations, reflecting the study’s interest in definitional use as an epistemic practice rather than in tracing conceptual originality. Coding proceeded inductively to identify recurring patterns. Following Patton’s (2002) principles of internal homogeneity and external heterogeneity, the resulting categories were iteratively refined so that elements grouped within a category shared a coherent conceptual meaning, while categories themselves remained clearly distinct. In practice, each category was designed to capture a single, internally consistent dimension of definitional variation, and overlapping or ambiguous codes were revised until clear boundaries were established. Because this review synthesises conceptual definitions rather than empirical outcomes or effect estimates, we did not conduct risk-of-bias assessments, reporting-bias assessments, or certainty-of-evidence evaluations (e.g., GRADE), which are typically applied in systematic reviews of quantitative outcome studies. Genealogical mapping was introduced as a complementary strategy: while the qualitative content analysis focuses on the internal structure of definitions, the genealogical analysis traces patterns of definitional reuse at the level of cited formulations rather than formal citation metrics. This process resulted in five analytical dimensions capturing distinct aspects of how conspiracy theories are conceptualised, as summarised in Table 2 below.

**Table 2.** Dimensions and categories of the qualitative analysis.

Dimension	Category	Analytical focus
Ontological	Discursive element; Mental product; Social action	What conspiracy theories are understood to be
Evaluative	Neutral-descriptive; Normative	Moral, social, or legitimacy judgments
Epistemic	Deficient; Neutral	Claims about knowledge quality or reasoning structure
Constitutive Features	Coalition; Contradicting; Intentionality; Malevolence; Power; Secrecy	Features treated as definitional components
Scope	Broad; Narrow; No scope	Domain and range of application

## Results

The definitions are organised along five dimensions: the ontological status attributed to conspiracy theories, the evaluative stance embedded in definitions, their epistemic characterisation, the constitutive features invoked, the patterns of co-occurrence among these elements, and the scope of phenomena to which conspiracy theories are applied. These dimensions correspond to analytically distinct yet complementary questions that definitions consistently address: what conspiracy theories are, how they are judged, how their knowledge claims are framed, which features are treated as constitutive, how those features combine in definitional formulations, and how broadly conspiratorial explanations are assumed to apply. For example, the analysis treated evaluative stance and epistemic characterisation as separate dimensions. Evaluative stance refers to moral judgments embedded in definitional statements, such as portraying conspiracy theories as harmful, pathological, or socially undesirable. Epistemic characterisation, by contrast, captures claims about the quality of knowledge or reasoning attributed to conspiracy theories, including references to weak evidence, resistance to falsification, or flawed inferential processes. Definitions were coded as epistemically deficient only when such claims were explicitly articulated in the definitional formulation itself, rather than inferred from the author’s broader argument or normative position.

### Ontological dimension

Definitions cluster around three ontological conceptualisations: conspiracy theories are defined as discursive entities, belief-based constructs, or social actions. Most conceptualise conspiracy theories as discursive constructions, such as narratives or explanatory accounts that connect events through assumptions of secret and intentional agency. A smaller subset defines conspiracy theories as belief-based phenomena, while a minority frames them as social actions involving coordinated collusion. Discursive definitions treat conspiracy theories primarily as stories or explanations rather than as psychological states or material practices. Keeley (1999, 116), for example, defines a conspiracy theory as ‘a proposed explanation of some historical event (or events) in terms of the significant causal agency of a relatively small group of persons—the conspirators—acting in secret’. Belief-based definitions instead conceptualise conspiracy theories as convictions through which individuals interpret social reality. Ernst and Fasce (2018, 54), for instance, use a definition of conspiracy theory as ‘a lay belief that attributes the ultimate cause of an event, or the concealment of an event from public knowledge, to a secret, unlawful, and malevolent plot by multiple actors working together’. Finally, a smaller group of definitions emphasises conspiracies as coordinated social actions by powerful actors, framing conspiracy theories as explanations of such plots. Wood, Douglas and Sutton (2012, 767), for example, use a definition of a conspiracy theory as ‘a proposed plot by powerful people or organizations working together in secret to accomplish some (usually sinister) goal’. The ontological emphasis on coordinated action and power is associated with differences in how conspiracy theories are evaluated across definitions.

### Evaluative dimension

The majority of definitions refrain from judging conspiracy theories as false, irrational, or harmful, instead presenting them as analytical objects. A smaller number characterise conspiracy theories as unfounded, implausible, or socially detrimental. Neutral-descriptive definitions emphasise analytical distance from pejorative meanings of conspiracy theories. Basham and Dentith (2016), for example, argue that conspiracy explanations should be assessed on a case-by-case evidentiary basis and reject the view that such explanations are inherently flawed. In contrast, normative definitions embed evaluative judgments, often portraying conspiracy theories as epistemically or socially problematic. Brotherton and French (2014, 238), for instance, use the following definition: ‘an unverified and relatively implausible allegation of conspiracy, claiming that significant events are the result of a secret plot carried out by a preternaturally sinister and powerful group of people’.

### Epistemic dimension

Only a minority of definitions address the epistemic status of conspiracy theories, and those that do tend to emphasise their deficiencies. Definitions addressing epistemic characteristics frequently describe conspiracy theories as resistant to falsification, reliant on weak evidence, or structured by flawed reasoning. Common formulations include references to ‘discounting contradictory evidence’, ‘self-insulating against disconfirmation’, and ‘fallacious logic’. They portray conspiracy theories as epistemically problematic forms of knowledge production, emphasising their internal reasoning processes rather than their empirical truth or falsity. By contrast, most definitions avoid epistemic judgments altogether, treating the validity of conspiracy theories as an empirical matter to be assessed individually rather than as a defining feature of the category itself. Beyond epistemic characterisation, definitions also vary in the specific attributes they identify as constitutive of conspiracy theories.

### Constitutive features

Conspiracy theories are typically defined through multiple features. Six recurring themes were identified: intentionality, coalition, secrecy, power, malevolence, and contradiction of official explanations. Intentionality is the most prevalent, with definitions portraying conspiracies as the outcome of agency exercised by individuals, groups, or institutions. Coalition frequently accompanies intentionality, emphasising coordinated action among actors, while secrecy appears prominently, referring either to hidden plans or to the clandestine nature of the actors involved. References to power and malevolent intent vary in emphasis across definitions, suggesting that they often function as complementary. This reveals that some formulations restrict themselves to a core constellation of intentional, coordinated secrecy, while others layer additional attributes such as power asymmetries or malevolent intent. A smaller number of definitions characterise conspiracy theories primarily through their opposition to official explanations, framing them as counter-hegemonic accounts. In rare cases, authors adopt minimalist formulations, defining conspiracy theories as theories that posit conspiracies (e.g., Peels, Kindermann, and Ranalli 2023). Most definitions combine several elements. In particular, intentionality, coalition, and secrecy form a recurrent core constellation around which other features are layered. By contrast, contradiction of dominant explanations is the only feature that sometimes appears on its own, indicating that for some authors epistemic opposition to authoritative accounts is treated as sufficient for classification. Harris (2022, 8), for example, defines conspiracy theories as ‘theories that allege conspiracies and conflict with the claims of relevant epistemic authorities, where epistemic authority is a matter of credentials and positions’. In summary, conspiracy theories are most often defined through interacting causal, structural, and normative features.

### Scope

Most definitions leave the scope of conspiratorial explanation unspecified, while a smaller subset refers to either broad or narrow domains. Many employ general terms such as event, phenomenon or situation, without clarifying the scale or significance of what is being explained. Others associate conspiracy theories with large-scale or consequential events, whereas only a minority restrict scope to specific domains. Definitions that specify scope often refer to significant social and political events or to extensive networks shaping social life, suggesting a broad domain of influence. By contrast, a small number of definitions delimit scope narrowly, focusing on particular issue areas. For example, Guidry et al. (2015: 5053) define conspiracy theories in relation to accusations surrounding vaccine-related adverse events: ‘Accusing a group or organization (e.g., the government, pharmaceutical companies, or doctors) of having caused or covered up vaccine adverse reactions or events’. All in all, most definitions adopt a flexible or open-ended notion of scope.

### Genealogical structure and central definitions

Definitions are organised around a small number of recurrent reference points. Figure 2 below maps reference relationships among the definitional statements in the dataset and shows that a substantial proportion of studies reuse, adapt, or cite earlier definitions. The network is organised around a limited number of highly central nodes, most prominently the definitions proposed by Keeley (1999), Coady (2003), Bale (2007), Sunstein and Vermeule (2009), and Douglas et al. (2019). In this analysis, the genealogical structure is used to identify definitions occupying strategic positions in the literature and not to quantify impact. This structure provides a basis for examining whether the qualitative dimensions identified in the broader analysis also characterise definitional practices at the core of the field.

**Figure 2.**
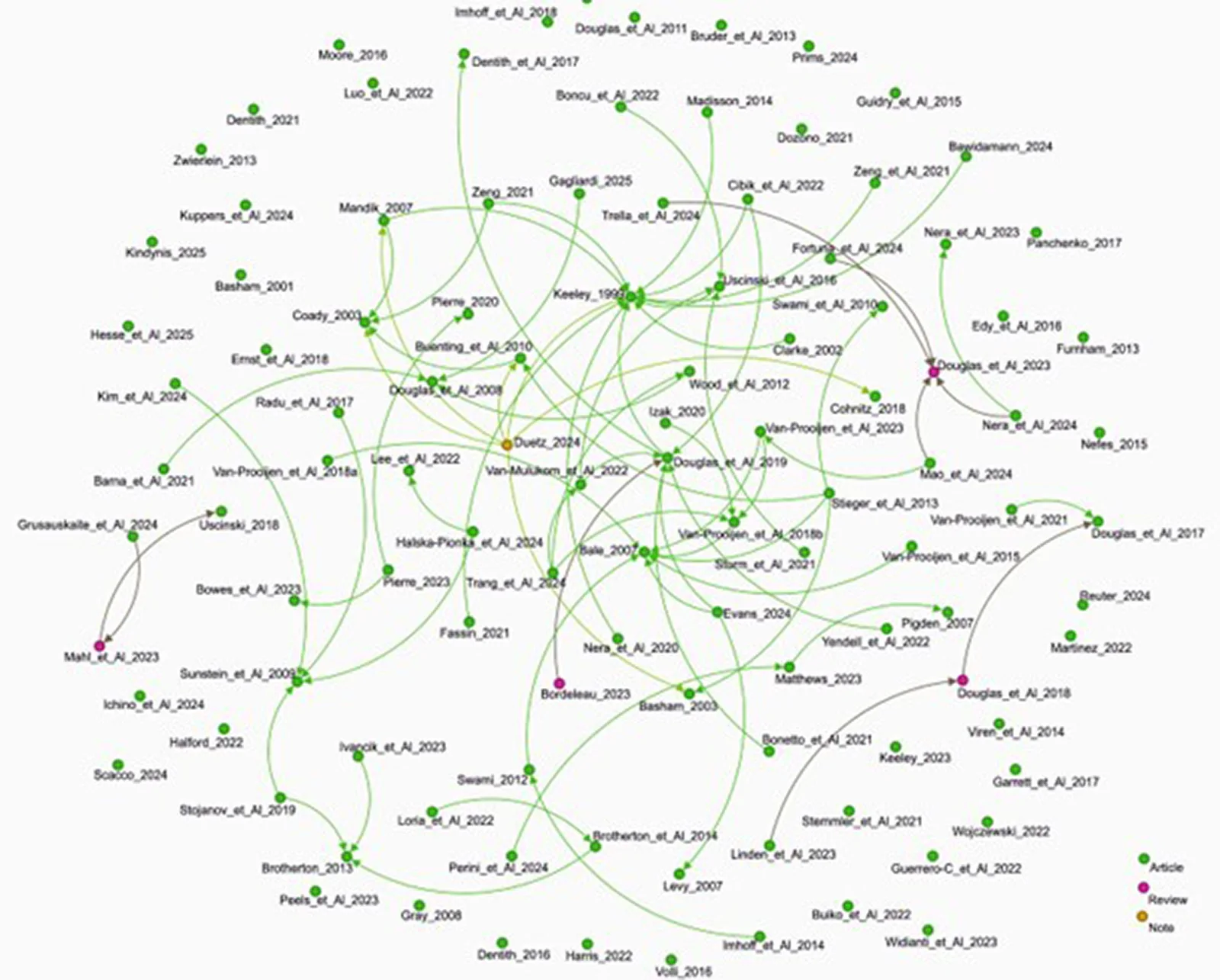
Citation relationships among CT definitions.

The central nodes correspond to a small set of widely used formulations. Keeley (1999) treats conspiracy theories as explanatory accounts that centre on covert coordination by a limited set of agents as the primary cause of events. Coady (2003) likewise conceptualises conspiracy theories as explanations of historical events in which conspiratorial agency plays a central causal role, adding that such explanations must posit a conspiracy aimed at bringing about the event and must conflict with an official account. Bale (2007) distinguishes between epistemically warranted conspiratorial explanations and what he terms bogus conspiracy theories: ‘Elaborate fantasies that purport to show that various sinister, powerful groups with evil intentions, operating behind the scenes, are secretly controlling the course of world events’. Sunstein and Vermeule (2009, 205) define them as ‘an effort to explain some event or practice by reference to the machinations of powerful people, who attempt to conceal their role (at least until their aims are accomplished)’. Douglas et al. (2019, 4) define conspiracy theories as ‘attempts to explain the ultimate causes of significant social and political events and circumstances with claims of secret plots by two or more powerful actors’. Although the definitions differ in emphasis, they share a focus on secret coordination, intentional agency, and explanatory or belief-based structures. The study examines how these definitions align and diverge across constitutive features, ontological, evaluative, epistemic, and scope-related dimensions.

Across these definitions, intentionality and secrecy recur: conspiracy theories are framed as accounts that attribute events or practices to deliberate, concealed coordination by multiple actors ( Keeley 1999; Coady 2003; Sunstein and Vermeule 2009; Douglas et al. 2019). Coalition is implicit in all cases. By contrast, power and malevolence are unevenly treated. Sunstein and Vermeule (2009) and Douglas et al. (2019) explicitly foreground power, whereas Keeley (1999) and Coady (2003) do not treat power asymmetries as a necessary defining condition. Malevolent intent is not required by Keeley (1999), Coady (2003), or Douglas et al. (2019). Bale’s (2007) contribution differs in kind: rather than offering a definition, he distinguishes between epistemically warranted conspiratorial explanations and what he terms bogus conspiracy theories, characterised by exaggerated scope, malevolence and weak evidential support. These patterns indicate that a shared core—intentional, secret coordination by multiple actors—anchors the genealogical centre of the field, while other elements such as power or epistemic quality are variably attached. Divergence across definitions therefore reflects differences in analytical emphasis rather than disagreement over what minimally constitutes a conspiracy theory. As subsequent studies draw selectively on these formulations, they inherit particular combinations of constitutive and evaluative elements.

Ontologically, the central definitions consistently frame conspiracy theories as explanatory accounts attributing events to concealed conspiratorial agency. Keeley (1999) and Coady (2003) frame conspiracy theories as explanatory accounts that attribute historical events to conspiratorial agency operating in secret. Douglas et al. (2019) likewise define conspiracy theories as attempts to explain the ultimate causes of significant social and political events by invoking secret plots among powerful actors, thereby also adopting an explanatory, discursive framing. Bale’s (2007) discussion treats conspiracy theories as explanations distinguished by their epistemic warrant. Sunstein and Vermeule (2009) characterise conspiracy theories as explanatory efforts that appeal to concealed machinations of powerful people.

This ontological convergence contrasts with greater variation in how the central definitions embed evaluative and epistemic judgments. Keeley (1999) and Coady (2003) adopt a neutral-descriptive stance and refrain from embedding general judgments about the epistemic quality, rationality, or social consequences of conspiracy theories. Douglas et al. (2019) likewise offer a descriptive definition focused on explanatory form without building evaluative or epistemic deficiency into the definition. Bale (2007) foregrounds epistemic status as a central concern. Sunstein and Vermeule (2009) embed negative evaluative and epistemic judgments, portraying conspiracy theories as systematically unreliable and socially harmful. These differences indicate that even among the most frequently reused definitions, there is no shared evaluative or epistemic stance. Instead, central definitions encode distinct analytical priorities, ranging from descriptive neutrality to normative critique.

Among the central definitions, there is substantial convergence in how broadly the scope of conspiracy theories is specified. Influential formulations by Keeley (1999), Coady (2003), and Douglas et al. (2019) define conspiracy theories in general terms, referring to events, or circumstances without delimiting specific empirical domains or issue areas. Bale’s (2007) intervention similarly operates at a general level by distinguishing between different kinds of conspiratorial explanations without restricting their application to particular fields. Sunstein and Vermeule’s (2009) definition also adopts a broad scope, despite being motivated by policy concerns, framing conspiracy theories as explanations of events or practices involving secret plots rather than as domain-specific phenomena. This pattern mirrors the wider corpus, where scope is rarely specified explicitly and conspiratorial explanation is treated as potentially applicable across diverse contexts. The persistence of broad or open-ended scope among the most frequently reused definitions implies that conceptual generality—rather than domain-specific precision—is stabilised in the core of the field, reinforcing both the portability and the indeterminacy of the concept.

## Discussion and conclusion

This study examined how conspiracy theories are defined in the academic literature and identified systematic conceptual diversity structured around recurring dimensions. Across 107 publications, definitions varied along ontological, evaluative, epistemic, structural, and scope-related dimensions. Most conceptualise conspiracy theories as discursive entities, adopt neutral-descriptive evaluative stances, avoid explicit epistemic judgments, and combine multiple constitutive features. At the same time, genealogical analysis shows that the most frequently used definitions converge ontologically while diverging in evaluative, epistemic, and constitutive-feature terms. Because these diverse formulations function as shared reference points, their repeated adoption through citation shapes conceptual plurality as the genealogical mapping demonstrates. Understanding definitional diversity as structurally reproduced helps to clarify why conceptual disagreement remains a defining feature of conspiracy theory research. By treating definitions as the unit of analysis, this review extends systematic review methodology beyond synthesising empirical findings to examining the conceptual infrastructures that structure research fields.

One of the central findings concerns the predominance of discursive ontological definitions combined with neutral-descriptive evaluative stances. Most definitions treat conspiracy theories as narratives or explanations and refrain from embedding normative judgments about their truth, irrationality, or harm. Importantly, the observed diversity is not reducible to disagreement over whether conspiracy theories are epistemically deficient, as many definitions that remain epistemically neutral nonetheless embed strong evaluative judgments, underscoring the relevance of separating epistemic characterisation from evaluative stance. This reflects a tendency to preserve analytical openness by separating scholarly concepts from everyday pejorative uses of ‘conspiracy theory’. Indeed, studies demonstrate that conspiracy theories may be deployed instrumentally in mainstream political settings rather than exclusively as expressions of irrationality ( Nefes 2014, 2015, 2026). By framing conspiracy theories as discursive objects, researchers could analyse their structure, circulation, and social functions without presupposing epistemic failure or moral deficiency.

Epistemic characterisation is a site of divergence. A minority of definitions describe conspiracy theories as epistemically deficient, while most avoid general claims about truth, rationality, or evidentiary standards. This reluctance seems to reflect an awareness of the risks of categorical judgment, including conceptual circularity and normative overreach. However, it also introduces analytical ambiguity, particularly for research designs that require clear conceptual boundaries between conspiracy theories and other explanatory forms. Together with the multidimensional structure and typically open-ended scope of most definitions, this ambiguity complicates comparison across studies and increases the risk of conceptual stretching.

The findings have broader implications. They show that more used definitions combine a shared core of constitutive features—most notably intentionality, secrecy, and coordination—with substantial variation in evaluative and epistemic orientation. At the same time, the analysis reveals a patterned mosaic of definitional architectures. While many formulations converge on a minimal core of intentional, secret coordination, others extend this by embedding assumptions about power asymmetries or malevolent intent, and a smaller subset treats opposition to dominant explanations as sufficient for classification. These alternative structural configurations represent distinct ways of constructing the category rather than marginal terminological differences. From a sociological perspective, this pattern suggests that definitional diversity is not a residual problem to be eliminated but a structured outcome of how academic fields stabilise concepts. The generalist–particularist divide is thus sustained not only by philosophical disagreement but by definitional practices that allow different research agendas to coexist under a shared label while embedding distinct assumptions about rationality, harm, and legitimacy.

Definitional diversity is not merely a conceptual issue but has implications for empirical operationalisation and measurement. When definitions embed evaluative or epistemic assumptions, these assumptions may shape survey instruments and interpretive frameworks. This underlines the importance of positing definitions explicitly rather than treating them as background assumptions. Furthermore, definitional dynamics become particularly consequential when concepts travel beyond academia. Institutional definitions—such as those used by the European Commission—often embed assumptions of inherent malevolence and epistemic deficiency, while the academic literature documents neutral and context-dependent definitions, including cases where conspiratorial narratives are framed as protective or order-preserving. When policy frameworks implicitly adopt narrow or normatively loaded definitions, they risk conflating analytical categorisation with moral judgment. Such selective uptake draws on particular academic lineages while obscuring the broader conceptual diversity of the field. Without awareness of this diversity, policy interventions may unintentionally reinforce conceptual ambiguity or politicise the category of conspiracy theory itself.

The findings should be interpreted in light of two main limitations. First, the analysis is restricted to English-language, peer-reviewed publications and focuses exclusively on explicit definitions. Second, many reviewed studies leave key dimensions—such as epistemic criteria or scope—underspecified. The first limitation may underrepresent influential implicit conceptualisations and non-English traditions. This was addressed through a transparent and systematic search strategy. The second reflects field-level constraints, and this was addressed by applying an inductive analytical framework that disentangles ontological, evaluative, epistemic, and structural dimensions. All in all, these limitations delimit the scope of inference without undermining the findings.

Nevertheless, the study makes contributions and points to directions for future research. By systematically reviewing an interdisciplinary corpus of definitions and mapping both their content and genealogical structure, the study shows that definitional diversity is reproduced through both analytical choices and referencing practices. Focusing on definitions themselves clarifies the conceptual foundations upon which empirical research, measurement strategies, and policy debates are built. Future research would benefit from greater reflexivity in definitional choices, explicit justification of analytical priorities, and clearer distinctions between ontological, evaluative, and epistemic considerations. Treating definitions as analytical tools whose suitability depends on research aims can enhance conceptual clarity, empirical rigour, and cumulative knowledge in the study of conspiracy theories.

## Ethics and consent

Ethical approval and consent were not required for this study. The research is based on the analysis of published academic literature and does not involve human participants, personal data, or identifiable information.

## Data Availability

The dataset generated and analysed in this study is publicly available in the DIGITAL.CSIC repository and can be accessed without restrictions. DIGITAL.CSIC: *Bibliographic data from publications with definitions of conspiracy theory and characterisation of the definitions.* https://doi.org/10.20350/digitalCSIC/17922 ( González-Albo, B. et al. 2025). The dataset contains definitions of “conspiracy theory” extracted from peer-reviewed academic publications written in English, together with bibliographic metadata and coded analytical dimensions used in this study, including:
- ontological assumptions - evaluative orientations - epistemic characterisations - constitutive features - conceptual scope Data are available under the terms of the Creative Commons Attribution 4.0 International license (CC-BY 4.0). No proprietary software was used in the analysis. Qualitative coding and analysis were conducted using standard text-processing and spreadsheet tools. PRISMA 2020 CHECKLIST is available in ZENODO repository with the following name: Prisma 2020 checklist with guidelines for submitting review reports for the article: “How conspiracy theories are defined”. DOI: https://doi.org/10.5281/zenodo.18956816. ( Martínez Escobar et al. 2026). Data are available under the terms of the Creative Commons Attribution 4.0 International license (CC-BY 4.0).
