# How conspiracy theories are defined: A systematic qualitative review of academic definitions

**DOI:** 10.12688/openreseurope.23324.2

**Published:** 2026-08-21

**Authors:** Türkay Salim Nefes, Javier Martínez Escobar, Borja González-Albo

**Affiliations:** 1 The Institute of Public Goods and Policies (IPP), Consejo Superior de Investigaciones Cientificas, Madrid, Community of Madrid, 28037, Spain; 2 Siyavuspasa Mah, Cigdem Sk, Mutlu Ap, TR-34182 Istanbul, Turkey; 3 Information Management Unit, Consejo Superior de Investigaciones Cientificas, Madrid, Community of Madrid, 28037, Spain

**Keywords:** Conspiracy theories; citation analysis; definitions; conceptual variation; qualitative analysis, systematic review.

## Abstract

**Background:**

Although conspiracy theories are studied across multiple disciplines, their definition remains unsettled. Despite a substantial body of empirical research, definitions have not been systematically analysed, leaving it unclear how conceptual variation shapes research, methods, and findings.

**Methods:**

Drawing on an original dataset of peer-reviewed publications, this study applies inductive qualitative content analysis to identify recurring dimensions along which academic definitions vary, including ontological assumptions, evaluative orientations, epistemic characterisations, constitutive features and scope. It further introduces a genealogical mapping of citation relationships to assess whether these dimensions structure more frequently reused definitions.

**Results:**

The qualitative content analysis finds that most definitions describe conspiracy theories as explanations and adopt a neutral-descriptive stance, with intentionality, coalition, and secrecy forming a recurrent core constellation of constitutive features. The genealogical mapping shows that frequently reused definitions share these features but differ in their evaluative and epistemic characterisation.

**Conclusions:**

Definitional diversity in conspiracy theory research is maintained through citations. Making definitional choices explicit and treating definitions as analytical tools rather than background assumptions can improve conceptual clarity, comparability, and cumulative research.

## Plain Language summary

Researchers do not all agree on what a “conspiracy theory” is. Studies use different definitions, but these differences are rarely examined systematically. Yet the way a concept is defined affects how research findings are interpreted and compared.

In this study, we reviewed peer-reviewed publications that explicitly define the term “conspiracy theory.” We examined each definition to identify recurring patterns. We looked at what conspiracy theories are described as, whether definitions include value judgments, how they treat knowledge and evidence, which features are considered essential, and how broadly the concept is applied.

We found that most definitions describe conspiracy theories as explanations involving secret coordination by multiple actors. However, they differ in important ways. Some definitions include negative judgments about truth or rationality, while others remain neutral. These differences also appear among the most frequently reused definitions and are repeated over time through citations.

Overall, there is no single shared academic definition of conspiracy theory. Instead, different approaches coexist. Making these differences explicit can improve clarity, transparency, and comparability in future research and in policy discussions that rely on academic concepts.

## Introduction

Recent scholarship notes that the academic literature lacks a standard definition of conspiracy theories despite increased empirical attention ( Nera and Schöpfer 2023; Uscinski and Enders 2023a). This lack of consensus has consequences beyond academia. For example, the European Commission defines conspiracy theories as beliefs involving secret manipulation by powerful actors with negative intent, embedding an assumption of inherent malevolence. Such framings exclude contexts in which conspiratorial narratives are interpreted as protective: In Turkey, the ‘deep state’ has often been portrayed as a covert security mechanism aimed at preserving political order not solely as a malign force ( Gürpınar 2019). The contrasting meanings attached to conspiracy theories show that the definitions shape which narratives are recognised as conspiratorial and how their social and political significance is interpreted. This motivates a closer examination of how they are defined.

Although definitional variations are acknowledged in research, they have not been examined systematically. Studies employ diverse definitions, ranging from neutral-descriptive formulations to normative ones, but this diversity tends to be treated as a background condition rather than as an object of analysis. It therefore remains unclear whether conceptual variation reflects substantive analytical differences or the uneven inheritance of earlier formulations. This study addresses this problem by analysing definitions in peer-reviewed articles through inductive qualitative content analysis and genealogical mapping of citation relationships. Combining these approaches allows us to assess whether conceptual diversity diminishes or persists at the core of the field. The review is guided by two research questions:

(1) How are conspiracy theories explicitly defined in peer-reviewed academic literature?
(2) Along which recurring conceptual dimensions does definitional variation occur, and how are these patterns reproduced through citation practices?

The article proceeds in four steps. First, it situates the study in the scholarship and highlights the lack of systematic analysis of definitions. Second, it describes the construction of the dataset and the inductive qualitative content analysis used to identify definitional dimensions, together with the genealogical mapping of citation relationships. Third, it presents the findings, detailing how definitions vary along ontological, evaluative, epistemic, constitutive-feature, and scope-related dimensions. Finally, it discusses the implications of these patterns for conceptual clarity and outlines potential directions for future studies.

## Defining conspiracy theories: Academic research and conceptual gaps

Research on conspiracy theories has expanded rapidly over the past two decades ( Rao and Greve 2024). Reviews document extensive work on the antecedents, correlates, and consequences of conspiracy beliefs, particularly in psychology, where scholars examined cognitive and affective processes such as uncertainty, threat perception, and distrust ( Douglas et al. 2019; Douglas and Sutton 2023). Other contributions situate conspiracy theories in relation to social and political topics such as democratic governance, epistemic authority, political communication, and cultural meaning-making, drawing on philosophy, political science, sociology, and communication studies ( Douglas et al. 2019). In these strands, conspiracy theories are treated as significant phenomena, linked to political mobilisation, institutional and interpersonal distrust, and misinformation dynamics. This literature generated substantial empirical findings and theoretical insights into why conspiracy theories emerge and how they operate in social life.

Despite this growth, definitions of conspiracy theories are often weakly specified or inconsistently applied. Mahl, Schäfer, and Zeng (2023) show that many studies rely on implicit or inherited understandings of conspiracy theories, leading to conceptual ambiguity. They show that definitions are frequently taken for granted, borrowed without reflection, or omitted altogether, even as the concept is operationalised in measurement and analysis. In parallel, Uscinski and Enders (2023b) argue that the absence of clear and consistently applied definitions allows conspiracy theory to function as a flexible and often subjective label rather than a stable analytical category, enabling evaluative judgments to enter implicitly through definitional practice. Moreover, conceptual ambiguity also arises from the frequent conflation of conspiracy theories with adjacent constructs such as conspiracy mentality or suspicion. Recent empirical work distinguishes between these related but analytically distinct phenomena, showing that dispositional conspiracy mentality and belief in specific conspiracy theories do not necessarily overlap and may have different attitudinal and behavioural correlates ( Nefes and Herreros 2026). Recognising such distinctions further underscores the importance of examining how conspiracy theory itself is defined in academic research.

When definitions are explicitly made, they vary considerably. Studies posit formulations, ranging from neutral explanatory accounts involving secret coordination to normative definitions that portray conspiracy theories as epistemically deficient or socially harmful ( Douglas et al. 2019). Philosophical scholarship has engaged most directly with such definitional and epistemic questions, particularly through debates over whether conspiracy theories are intrinsically suspect or should instead be assessed on a case-by-case basis ( Clarke 2002; Coady 2006; Dentith 2016; Keeley 1999). However, this work remains largely conceptual. Valuable contributions proposed, refined, or criticised definitions in relation to broader epistemic commitments. Consequently, definitional plurality is acknowledged but typically illustrated through a small number of canonical examples rather than systematically. Overall, the scholarship illustrates that definitional work is ubiquitous and weakly documented. What is missing is a systematic map of this definitional landscape—one that captures how conspiracy theories are conceptualised and how specific formulations are reproduced over time.

Disagreements over how conspiracy theories should be evaluated are closely tied to how they are defined, and these disagreements can be approached from a sociological perspective concerned with the construction of concepts. Particularist approaches argue that conspiracy theories should be assessed on a case-by-case basis and resist treating them as a unified category, typically favouring broad and neutral definitions centred on secret coordination ( Coady 2006). Generalist approaches, by contrast, maintain that conspiracy theories can be evaluated as a class and therefore rely on more restrictive definitions that foreground shared epistemic or normative deficiencies ( Harris 2022). From a sociological standpoint, these positions reflect competing ways of constructing the object of inquiry. Definitions do not simply describe conspiracy theories; they help constitute them as a category of knowledge. As Thomas and Thomas (1928) posited, when people define situations as real, those definitions have real consequences. Building on this insight, the present study shifts attention from how conspiracy theories ought to be evaluated to how they are actually defined. Treating definitions as socially situated practices provides a basis for examining their epistemic and practical consequences and understanding how conceptual choices shape the future trajectory of conspiracy theory research as well as policies.

## Methods

Data were collected by the three authors using a systematic bibliographic search in three major databases: Web of Science (WoS), Scopus, and OpenAlex. Databases were last searched in February 2025. The search strategy targeted publications defining or conceptualising conspiracy theories using two term sets: ( *conspira* + *theory* + (defin OR concept)); ( *conspiracism* + (defin OR concept)). Searches were limited to peer-reviewed articles, review articles, and notes published in English. Using multiple databases reduces disciplinary and publication bias and increases the likelihood of capturing influential contributions. Because database search options differ, proximity operators were applied in WoS and Scopus, whereas OpenAlex required broader Boolean combinations. This resulted in a higher volume of initial records in OpenAlex, which necessitated additional manual screening to reduce informational noise. The database showing, search logic, and retrieval results are reported in Table 1 below, which underpins the subsequent screening process.

**Table 1.** Search procedure for obtaining articles with a CT definition.

	WoS	Scopus	OpenAlex
Procedure	The databases used: Science Citation Index, Social Science Citation Index, Arts & Humanities Citation Index, and Emerging Science Citation Index. In this case, considering the following sets – a) conspira*; b) theory; c) (defin* or concept*); d) conspiracism; e) (defin* or concept*)- the search strategy establishes that terms a) and b) can be found within a maximum of three words of each other in the bibliographic fields. This set can be separated by a maximum of three words from c). Similarly, and d) and e) must be within a maximum of three words of each other. The search fields are the title, abstract, and keywords.	The same search strategy is used and with the same restrictions as for WoS.	Because the system does not allow the use of proximity operators between terms neither truncations, the single terms were considered for the topic definition, combining them with boolean operators. This results in greater informational noise in the results obtained, so it was necessary to perform an initial screening of the documents obtained. Therefore, two search strategies were used against the title and abstract fields, although they were combined into a single one for the database search: 1. (Conspiracy OR Conspiracist OR Conspiratorial) AND (theory) AND (definition OR define OR concept OR conceptualise). 2. (Conspiracism) AND (Theory) AND (definition OR define OR concept OR conceptualise).
Results	105 documents	154 documents	1638 initial documents; 159 potentially relevant

Retrieved records were consolidated and screened using eligibility criteria. All records were merged into a single database and deduplicated primarily using Digital Object Identifiers (DOIs). Where DOIs were unavailable, manual matching was conducted using title, author(s), journal, and year of publication, and the source database was retained using dummy variables. Eligibility was determined through criterion sampling ( Patton 2002). To be included in the analysis, a publication had to contain an explicit and author-endorsed definition of conspiracy theory. Publications referring to conspiracy theories only indirectly—through examples, descriptive discussions, or alternative labels such as *conspiracy narrative* or *conspiracy belief*—were excluded from the main corpus but reviewed separately to inform reflections on conceptual boundaries. Following initial screening, snowball sampling was applied by examining references cited in the included definitions to identify additional publications with definitions. This iterative process yielded an analytical corpus, the construction of which is summarised in Figure 1 below. Titles and abstracts were screened independently by two reviewers, and full-text articles were subsequently assessed for eligibility through discussion among the authors until consensus was reached.

**Figure 1.**
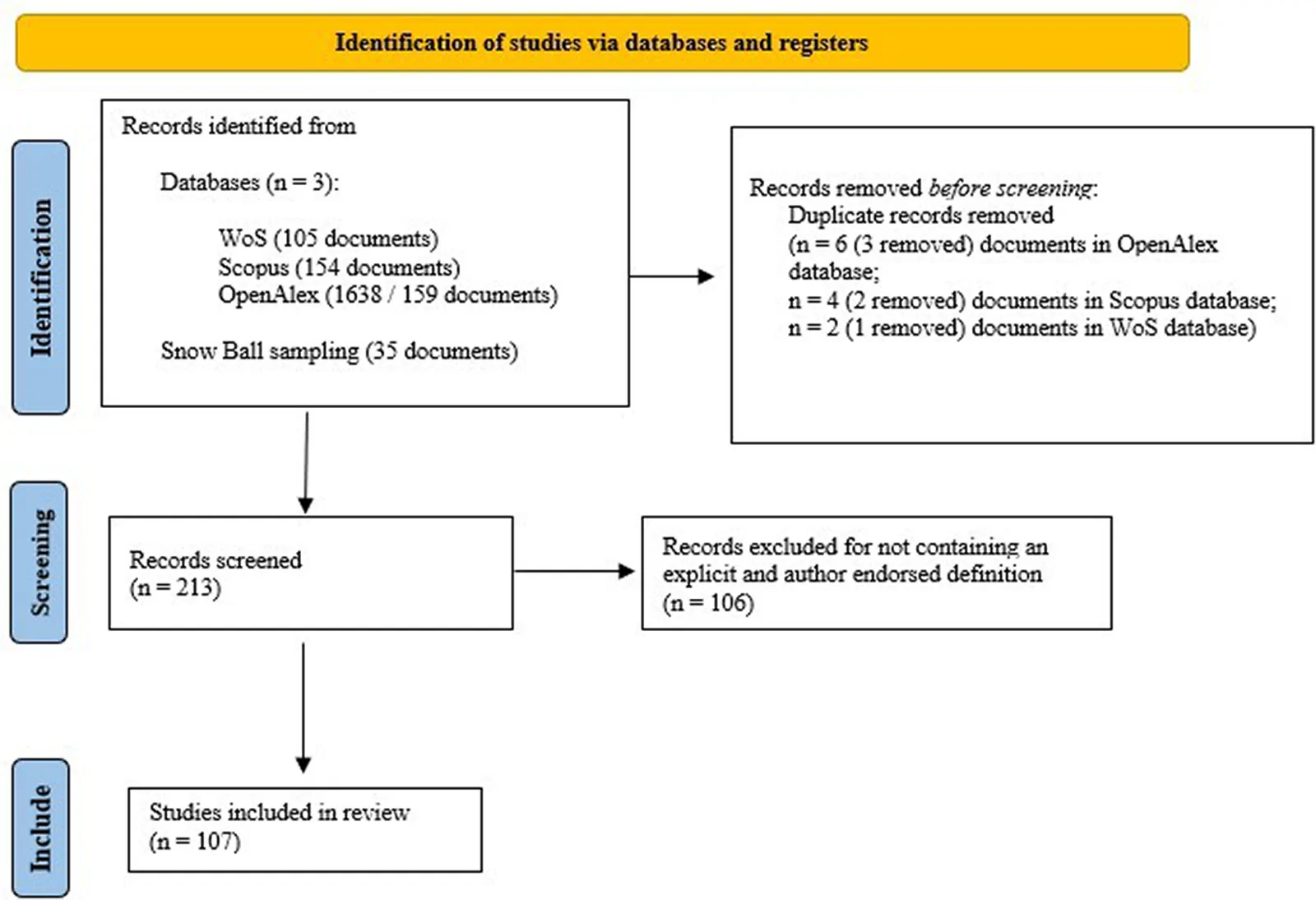
Flowchart of Identification, Screening, and Inclusion of Studies.

The final dataset ( González-Albo, Martínez Escobar and Nefes 2025) was analysed using qualitative content analysis. Data extraction and coding were conducted by the authors, with categories iteratively refined through discussion until consensus was reached. For each publication, the passage in which *conspiracy theory* was explicitly defined was treated as the unit of analysis. Identical or closely related formulations were retained as separate observations, reflecting the study’s interest in definitional use as an epistemic practice rather than in tracing conceptual originality. Coding proceeded inductively to identify recurring patterns. Following Patton’s (2002) principles of internal homogeneity and external heterogeneity, the resulting categories were iteratively refined so that elements grouped within a category shared a coherent conceptual meaning, while categories themselves remained clearly distinct. In practice, each category was designed to capture a single, internally consistent dimension of definitional variation, and overlapping or ambiguous codes were revised until clear boundaries were established. Because this review synthesises conceptual definitions rather than empirical outcomes or effect estimates, we did not conduct risk-of-bias assessments, reporting-bias assessments, or certainty-of-evidence evaluations (e.g., GRADE), which are typically applied in systematic reviews of quantitative outcome studies. Genealogical mapping was introduced as a complementary strategy: while the qualitative content analysis focuses on the internal structure of definitions, the genealogical analysis traces patterns of definitional reuse at the level of cited formulations rather than formal citation metrics. The network was constructed from citation relationships between definitions. Centrality was assessed using in-degree centrality, based on the number of definitions in the corpus citing each definition. The qualitative content analysis resulted in five analytical dimensions capturing distinct aspects of how conspiracy theories are conceptualised, as summarised in Table 2 below.

**Table 2.** Dimensions and categories of the qualitative analysis.

Dimension	Category	Analytical focus
Ontological	Explanatory/discursive object; Mental product/belief; Social action/practice	What conspiracy theories are understood to be
Evaluative	Neutral-descriptive; Normative	Moral, social, or legitimacy judgments
Epistemic	Deficient; Neutral	Claims about knowledge quality or reasoning structure
Constitutive Features	Coalition; Contradicting; Intentionality; Malevolence; Power; Secrecy; No elements	Features treated as definitional components
Scope of the Explained Phenomenon	Broad; Narrow; Unspecified	Domain and range of application

## Results

The definitions are organised along five dimensions: the ontological status attributed to conspiracy theories, the evaluative stance embedded in definitions, their epistemic characterisation, the constitutive features invoked and the scope of phenomena to which conspiracy theories are applied. These dimensions correspond to analytically distinct yet complementary questions that definitions consistently address: what conspiracy theories are, how they are judged, how their knowledge claims are framed, which features are treated as constitutive, how those features combine in definitional formulations, and how broadly conspiratorial explanations are assumed to apply. For example, the analysis treated evaluative stance and epistemic characterisation as separate dimensions. Evaluative stance refers to moral judgments embedded in definitional statements, such as portraying conspiracy theories as harmful, pathological, or socially undesirable. Epistemic characterisation, by contrast, captures claims about the quality of knowledge or reasoning attributed to conspiracy theories, including references to weak evidence, resistance to falsification, or flawed inferential processes. Definitions were coded as epistemically deficient only when such claims were explicitly articulated in the definitional formulation itself, rather than inferred from the author’s broader argument or normative position.

Table 3 below reports the absolute frequencies and percentages of the categories identified across the dimensions. Each definition was assigned to the category that best captured its primary or most salient formulation. Constitutive features are not mutually exclusive, as individual definitions may contain more than one feature.

**Table 3.** Prevalence of categories across definitional dimensions (N = 107).

Dimension	Category	N	%
Ontological	Explanatory/discursive object	85	79.4
	Mental product/belief	19	17.8
	Social action/practice	3	2.8
Evaluative	Neutral-descriptive	95	88.8
	Normative	12	11.2
Epistemic	Neutral	97	90.7
	Deficient	10	9.3
Constitutive features	Intentionality	90	84.1
	Coalition	80	74.8
	Secrecy	77	72
	Power	44	41.1
	Malevolence	39	36.4
	Contradicting	19	17.8
	No elements	12	11.2
Scope of explained phenomenon	Unspecified	74	69.2
	Broad	32	29.9
	Narrow	1	0.9

### Ontological dimension

Definitions cluster around three ontological conceptualisations. Most define conspiracy theories as explanatory or discursive objects, such as explanations, theories, claims, narratives, allegations, or interpretations (79%). A smaller group defines them as beliefs or other mental constructs (18%), while only 3% formulate them primarily as social actions or communicative practices. The distinction concerns what the definition presents the conspiracy theory itself as, not the actions attributed to the alleged conspirators. Thus, a definition describing a conspiracy theory as an explanation of secret coordinated action is classified as an explanatory or discursive object. Kindynis (2025), for example, defines conspiracy theories as ‘accounts or narratives that allege intrigue behind political or historical events’. Belief-based definitions instead identify the conspiracy theory itself as a construct. Ernst and Fasce (2018, 54), for instance, define it as ‘a lay belief that attributes the ultimate cause of an event, or the concealment of an event from public knowledge, to a secret, unlawful, and malevolent plot by multiple actors working together’. Social-action formulations are rare. Gray (2008, 159), for example, defines conspiracy theory or conspiracism as ‘the act of developing and sustaining a discourse’, thereby locating the phenomenon in a communicative practice rather than in the explanatory content or belief alone.

### Evaluative dimension

The majority of definitions refrain from embedding explicit negative evaluations, instead presenting conspiracy theories as analytical objects. A smaller number characterise conspiracy theories in pejorative or socially negative terms. Neutral-descriptive definitions emphasise analytical distance from pejorative meanings of conspiracy theories. Basham and Dentith (2016), for example, argue that conspiracy explanations should be assessed on a case-by-case evidentiary basis and reject the view that such explanations are inherently flawed. In contrast, normative definitions embed evaluative judgments, often portraying conspiracy theories as socially problematic or otherwise undesirable. Brotherton and French (2014, 238), for instance, use the following definition: ‘an unverified and relatively implausible allegation of conspiracy, claiming that significant events are the result of a secret plot carried out by a preternaturally sinister and powerful group of people’.

### Epistemic dimension

Only a minority of definitions address the epistemic status of conspiracy theories, and those that do tend to emphasise their deficiencies. Definitions addressing epistemic characteristics frequently describe conspiracy theories as resistant to falsification, reliant on weak evidence, or structured by flawed reasoning. Common formulations characterise conspiracy theories as ‘epistemically self-insulating against disconfirmation’ ( Brotherton 2013, 9) or as relying on ‘weak, flawed, or fallacious logic’ ( Gray 2008, 159). They portray conspiracy theories as epistemically problematic forms of knowledge production, emphasising their internal reasoning processes rather than their empirical truth or falsity. By contrast, most definitions avoid epistemic judgments altogether, treating the validity of conspiracy theories as an empirical matter to be assessed individually rather than as a defining feature of the category itself. Beyond epistemic characterisation, definitions also vary in the specific attributes they identify as constitutive of conspiracy theories.

### Constitutive features

Conspiracy theories are typically defined through multiple features. Six recurring themes were identified: intentionality, coalition, secrecy, power, malevolence, and contradiction of official explanations. Intentionality is the most prevalent, with definitions portraying conspiracies as the outcome of agency exercised by individuals, groups, or institutions. Coalition frequently accompanies intentionality, emphasising coordinated action among actors, while secrecy appears prominently, referring either to hidden plans or to the clandestine nature of the actors involved. References to power and malevolent intent vary in emphasis across definitions, suggesting that they often function as complementary. A smaller number of definitions characterise conspiracy theories primarily through their opposition to official explanations, framing them as counter-hegemonic accounts. In rare cases, authors adopt minimalist formulations, defining conspiracy theories as theories that posit conspiracies (e.g., Peels, Kindermann, and Ranalli 2023).

Most definitions combine several elements. In particular, intentionality, coalition, and secrecy form a recurrent core constellation around which other features are layered. However, some of these features may be prominent partly because they are already contained in the underlying concept of conspiracy rather than because authors introduce them as additional criteria for defining conspiracy theory. This is particularly clear for coalition and coordination: the Latin conspirare literally means ‘to breathe together’ and came to denote acting or agreeing together, while earlier English usage could similarly refer to harmonious combination. Sidney’s Arcadia, for example, describes ‘the conspiracy of her several graces, held best together to make one perfect figure of beauty’ ( Sidney [1590] 1907, 497). Definitions of conspiracy theory may then add further criteria that are not inherent in the concept of conspiracy itself. Contradiction of dominant or authoritative explanations is one such criterion. This may help explain why it sometimes appears without the other constitutive features: unlike coalition and coordination, it can independently serve to distinguish a conspiracy theory from other claims about conspiracies. For some authors, such opposition to authoritative accounts is sufficient for classification. Harris (2022, 8), for example, defines conspiracy theories as ‘theories that allege conspiracies and conflict with the claims of relevant epistemic authorities, where epistemic authority is a matter of credentials and positions’. In summary, conspiracy theories are often defined through interacting causal, structural, and evaluative features.

### Scope of the explained phenomenon

Definitions also vary in whether they specify the domain or scale of the phenomenon that a conspiracy theory seeks to explain. Most definitions leave this unspecified, employing general terms such as event, phenomenon, or situation without defining its scale or social significance. A smaller subset associates conspiracy theories with broad or consequential social and political events, while only a minority restricts them to particular domains or issue areas. The dimension therefore concerns the range of phenomena to which a definition applies rather than the conceptual breadth of conspiracy theory itself. For example, Guidry et al. (2015, 5053) define conspiracy theories specifically in relation to vaccine-related adverse events: ‘Accusing a group or organization (e.g., the government, pharmaceutical companies, or doctors) of having caused or covered up vaccine adverse reactions or events’.

### Genealogical structure and central definitions

Definitions are organised around a small number of recurrent reference points. Figure 2 below maps reference relationships among the definitional statements in the dataset and shows that a substantial proportion of studies reuse, adapt, or cite earlier definitions. The network is organised around a limited number of highly central nodes, most prominently the definitions proposed by Keeley (1999), Coady (2003), Bale (2007), Sunstein and Vermeule (2009), and Douglas et al. (2019). The genealogical analysis identifies definitions occupying central positions in this network, treating centrality as a measure of definitional relationships rather than broader citation impact. This structure provides a basis for examining whether the qualitative dimensions identified in the broader analysis also characterise definitional practices at the core of the field.

**Figure 2.**
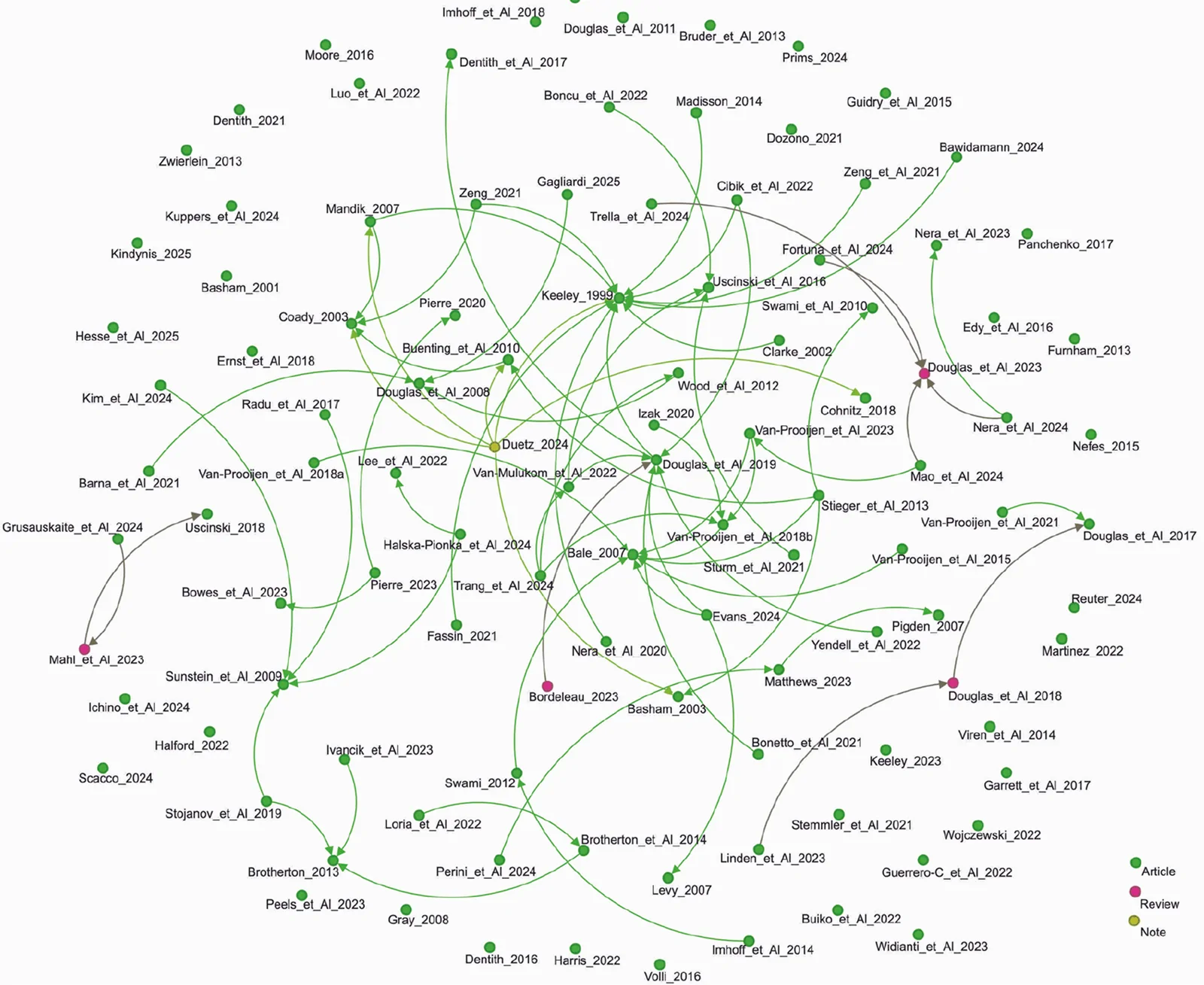
Citation relationships among CT definitions.

The central nodes correspond to a small set of widely used formulations. Keeley (1999) treats conspiracy theories as explanatory accounts that centre on covert coordination by a limited set of agents as the primary cause of events. Coady (2003) likewise conceptualises conspiracy theories as explanations of historical events in which conspiratorial agency plays a central causal role, adding that such explanations must posit a conspiracy aimed at bringing about the event and must conflict with an official account. Bale (2007) distinguishes between epistemically warranted conspiratorial explanations and what he terms bogus conspiracy theories: ‘Elaborate fantasies that purport to show that various sinister, powerful groups with evil intentions, operating behind the scenes, are secretly controlling the course of world events’. Sunstein and Vermeule (2009, 205) define them as ‘an effort to explain some event or practice by reference to the machinations of powerful people, who attempt to conceal their role (at least until their aims are accomplished)’. Douglas et al. (2019, 4) define conspiracy theories as ‘attempts to explain the ultimate causes of significant social and political events and circumstances with claims of secret plots by two or more powerful actors’.

Across these definitions, intentionality and secrecy recur: conspiracy theories are framed as accounts that attribute events or practices to deliberate, concealed coordination by multiple actors ( Keeley 1999; Coady 2003; Sunstein and Vermeule 2009; Douglas et al. 2019). Coalition is implicit in all cases. By contrast, power and malevolence are unevenly treated. Sunstein and Vermeule (2009) and Douglas et al. (2019) explicitly foreground power, whereas Keeley (1999) and Coady (2003) do not treat power asymmetries as a necessary defining condition. Malevolent intent is not required by Keeley (1999), Coady (2003), or Douglas et al. (2019). Keeley (1999) also distinguishes conspiracy theories as a general category from ‘unwarranted conspiracy theories’. In other words, being epistemically unwarranted is not part of his definition of a conspiracy theory; it is a characteristic that may apply to particular conspiracy theories. Bale (2007) makes a comparable distinction by separating epistemically warranted conspiratorial explanations from what he terms bogus conspiracy theories, which are characterised by exaggerated scope, malevolence, and weak evidential support. Both approaches therefore distinguish what makes an account a conspiracy theory from the separate question of whether that account is epistemically warranted. These patterns indicate that a shared core—intentional, secret coordination by multiple actors—anchors the genealogical centre of the field, while other elements such as power or epistemic quality are variably attached. Divergence across definitions therefore reflects differences in analytical emphasis rather than disagreement over what minimally constitutes a conspiracy theory. As subsequent studies draw selectively on these formulations, they inherit particular combinations of constitutive and evaluative elements.

Ontologically, these central definitions consistently frame conspiracy theories as explanatory accounts rather than as beliefs or social practices. This ontological convergence contrasts with some variation in how the central definitions treat evaluative and epistemic judgments. Keeley (1999) and Coady (2003) adopt a neutral-descriptive stance and refrain from embedding judgments about the epistemic quality or consequences of conspiracy theories. Douglas et al. (2019) likewise offer a descriptive definition focused on explanatory form without building evaluative or epistemic deficiency into the definition. Sunstein and Vermeule (2009) also use a neutral-descriptive definition, despite adopting a critical position towards conspiracy theories in their broader argument. Their definition identifies conspiracy theories through their explanatory form and reference to the concealed actions of powerful actors, without treating falsity, irrationality, or epistemic deficiency as criteria. This illustrates how the evaluative content of an explicit definition may differ from the broader assumptions surrounding its use. Bale (2007), by contrast, foregrounds epistemic status by distinguishing warranted conspiratorial explanations from bogus conspiracy theories.

Among the central definitions, there is substantial convergence in how broadly the scope of conspiracy theories is specified. Influential formulations by Keeley (1999), Coady (2003), and Douglas et al. (2019) define conspiracy theories in general terms, referring to events, or circumstances without delimiting specific empirical domains or issue areas. Bale’s (2007) intervention similarly operates at a general level by distinguishing between different kinds of conspiratorial explanations without restricting their application to particular fields. Sunstein and Vermeule’s (2009) definition also adopts a broad scope, despite being motivated by policy concerns, framing conspiracy theories as explanations of events or practices involving secret plots rather than as domain-specific phenomena. This pattern mirrors the wider corpus, where scope is rarely specified explicitly and conspiratorial explanation is treated as potentially applicable across diverse contexts. The persistence of broad or open-ended scope among the most frequently reused definitions implies that conceptual generality—rather than domain-specific precision—is stabilised in the core of the field, reinforcing both the portability and the indeterminacy of the concept.

## Discussion and conclusion

This study examined how conspiracy theories are defined in the academic literature and identified systematic conceptual diversity structured around recurring dimensions. Across 107 publications, definitions varied along ontological, evaluative, epistemic, constitutive-feature, and scope-related dimensions. Most conceptualise conspiracy theories as discursive entities, adopt neutral-descriptive evaluative stances, avoid explicit epistemic judgments, and combine multiple constitutive features. At the same time, genealogical analysis shows that the most frequently used definitions converge ontologically while diverging in evaluative, epistemic, and constitutive-feature terms. Because these diverse formulations function as shared reference points, their repeated adoption through citation shapes conceptual plurality as the genealogical mapping demonstrates. Understanding definitional diversity as structurally reproduced helps to clarify why conceptual disagreement remains a defining feature of conspiracy theory research. By treating definitions as the unit of analysis, this review extends systematic review methodology beyond synthesising empirical findings to examining the conceptual infrastructures that structure research fields.

One of the central findings concerns the predominance of discursive ontological definitions combined with neutral-descriptive evaluative stances. Most definitions treat conspiracy theories as narratives or explanations and refrain from embedding explicit evaluative judgments about their harm or legitimacy. The observed diversity is not reducible to disagreement over whether conspiracy theories are epistemically deficient, as some definitions that remain epistemically neutral nonetheless embed evaluative judgments, underscoring the relevance of separating epistemic characterisation from evaluative stance. This reflects a tendency to preserve analytical openness by separating scholarly concepts from everyday pejorative uses of ‘conspiracy theory’. Indeed, studies demonstrate that conspiracy theories may be deployed instrumentally in mainstream political settings rather than exclusively as expressions of irrationality ( Nefes 2014, 2015, 2017, 2026). By framing conspiracy theories as discursive objects, researchers could analyse their structure, circulation, and social functions without presupposing epistemic failure or moral deficiency.

Epistemic characterisation is a site of divergence. A minority of definitions describe conspiracy theories as epistemically deficient, while most avoid general claims about truth, rationality, or evidentiary standards. This reluctance seems to reflect an awareness of the risks of categorical judgment, including conceptual circularity and normative overreach. However, it also introduces analytical ambiguity, particularly for research designs that require clear conceptual boundaries between conspiracy theories and other explanatory forms. Together with the multidimensional structure and typically open-ended scope of most definitions, this ambiguity complicates comparison across studies and increases the risk of conceptual stretching. A related issue concerns the relationship between definitional breadth and case selection. Nera and Schöpfer (2023) observe that researchers may adopt definitions broad enough to encompass conspiracy claims generally while investigating a more restricted set of cases, particularly those regarded as unwarranted. As a result, conclusions derived from these cases may not extend to the full range of phenomena included by the definition. This discrepancy has implications for theoretical inference, since findings attributed to conspiracy theories generally may apply more narrowly to particular types of conspiracy theory. The present review does not examine empirical case selection, but the definitional variation identified here suggests that future research should consider whether the cases studied correspond to the boundaries established by the definitions employed.

Definitional choices have consequences for how conspiracy theories are studied. Our findings show that most definitions are neutral-descriptive, while others add evaluative or epistemic criteria or adopt more minimalist formulations. Neutral-descriptive and minimalist definitions leave more room for assessing individual cases, but provide fewer criteria for operationalising the concept and distinguishing conspiracy theories from related concepts such as conspiracy mentality or rumour. More restrictive definitions provide clearer boundaries, but make classification partly dependent on evaluative or epistemic assumptions about the phenomena being studied. Broad definitions enhance portability and comparison across domains, whereas narrower definitions improve precision at the expense of generalisability. Definitions should therefore be made explicit and justified according to the aims of the study.

The different definitional configurations identified here represent distinct ways of constructing the category rather than marginal terminological differences. From a sociological perspective, definitional diversity appears as a structured outcome of how academic fields stabilise concepts. The generalist–particularist divide is therefore sustained not only by philosophical disagreement but also by definitional practices that allow different research agendas to coexist under a shared label while embedding distinct assumptions about rationality, harm, and legitimacy.

Definitional diversity is not merely a conceptual issue but has implications for empirical operationalisation and measurement. When definitions embed evaluative or epistemic assumptions, these assumptions may shape survey instruments and interpretive frameworks. This underlines the importance of positing definitions explicitly rather than treating them as background assumptions. Furthermore, definitional dynamics become particularly consequential when concepts travel beyond academia. Institutional definitions—such as those used by the European Commission—often embed assumptions of inherent malevolence and epistemic deficiency, while the academic literature documents neutral and context-dependent definitions, including cases where conspiratorial narratives are framed as protective or order-preserving. When policy frameworks implicitly adopt narrow or normatively loaded definitions, they risk conflating analytical categorisation with moral judgment. Such selective uptake draws on particular academic lineages while obscuring the broader conceptual diversity of the field. Without awareness of this diversity, policy interventions may unintentionally reinforce conceptual ambiguity or politicise the category of conspiracy theory itself.

The findings should be interpreted in light of two main limitations. First, the analysis is restricted to English-language, peer-reviewed publications and focuses exclusively on explicit definitions. This may underrepresent influential implicit conceptualisations and non-English traditions. We first conducted broad searches across three major bibliographic databases. Snowball sampling was then used as a complementary method to identify additional definitions not captured by the database searches. Because this procedure follows existing citation relationships, it may underrepresent relevant definitions that are less frequently cited or weakly connected to established citation networks. Second, many reviewed studies leave key dimensions—such as epistemic criteria or scope—underspecified. This reflects field-level constraints and was addressed by applying an inductive analytical framework that disentangles ontological, evaluative, epistemic, constitutive-feature, and scope dimensions. All in all, these limitations delimit the scope of inference without undermining the findings.

Nevertheless, the study makes contributions and points to directions for future research. By systematically reviewing an interdisciplinary corpus of definitions and mapping both their content and genealogical structure, the study shows that definitional diversity is reproduced through both analytical choices and referencing practices. Focusing on definitions themselves clarifies the conceptual foundations upon which empirical research, measurement strategies, and policy debates are built. Future research would benefit from greater reflexivity in definitional choices, explicit justification of analytical priorities, and clearer distinctions between ontological, evaluative, and epistemic considerations. Treating definitions as analytical tools whose suitability depends on research aims can enhance conceptual clarity, empirical rigour, and cumulative knowledge in the study of conspiracy theories.

## Ethics and consent

Ethical approval and consent were not required for this study. The research is based on the analysis of published academic literature and does not involve human participants, personal data, or identifiable information.

## Data Availability

The dataset generated and analysed in this study is publicly available in the DIGITAL.CSIC repository and can be accessed without restrictions. The repository supports versioning of datasets, with updated versions assigned new DOIs. Repository: DIGITAL.CSIC DOI: https://doi.org/10.20350/digitalCSIC/17922 Dataset citation: González-Albo, B., Martínez Escobar, J., & Nefes, T. S. (2025). Bibliographic data from publications with definitions of conspiracy theory and characterisation of the definitions. DIGITAL.CSIC. https://doi.org/10.20350/digitalCSIC/17922 The dataset contains definitions of “conspiracy theory” extracted from peer-reviewed academic publications written in English, together with bibliographic metadata and coded analytical dimensions used in this study, including:
• ontological assumptions • evaluative orientations • epistemic characterisations • constitutive features • conceptual scope The dataset is available under the terms of the Creative Commons Attribution 4.0 International (CC BY 4.0) license. No proprietary software was used in the analysis. Qualitative coding and analysis were conducted using standard text-processing and spreadsheet tools. PRISMA 2020 CHECKLIST is available in ZENODO repository with the following name: Prisma 2020 checklist with guidelines for submitting review reports for the article: “How conspiracy theories are defined”. DOI: https://doi.org/10.5281/zenodo.18956816. Checklist citation: Martínez Escobar, J., Nefes, T. S., & González-Albo, B. (2026). Prisma 2020 checklist with guidelines for submitting review reports for the article: “How conspiracy theories are defined”. Zenodo. https://doi.org/10.5281/zenodo.18956816 This systematic review followed the PRISMA 2020 reporting guidelines. The completed PRISMA checklist is provided in a public repository and cited in the Data Availability statement. The review was not preregistered and no protocol was prepared.
