# Supplementary material for: How conspiracy theories are defined: A systematic qualitative review of academic definitions
Source: Open Res Eur. 2026 Oct 15;6:155. Originally published 2026 May 8. [Version 3] doi: 10.12688/openreseurope.23324.3 (PMC13631454; doi:10.12688/openreseurope.23324.3)
Supplement: Peer review history

# Reviewer report: Reviewer response for version 2

**DOI:** 10.21956/openreseurope.27009.r79733

**Published:** 2026-10-01

**Authors:** Dr. Anne Küppers

**Affiliations:** 1 University of Jena, Jena, Germany

The article performs a systematic review of conspiracy theories in peer-reviewed academic work (following PRISMA guidelines). It tracks commonalities and differences in conceptualization along 5 core dimensions. Further, a genealogical mapping traces citation relationships.

 I believe that the article will be extremely valuable to conspiracy research across disciplines. I particularly liked the attempt to move beyond simply listing existing definitions and to instead identify common dimensions along which definitions align or differ. Also, the dataset is another major contribution of the paper. Having a systematic collection of definitions used in peer-reviwed academic work, together with the information on the conceptual characteristics of each definition could be extremely useful to understand how conceptual and evaluative assumptions about CTs  feed into their operationalization (e.g. as survey items/batteries) and measurement in empirical research.

 Thus, overall, I think this is a already very strong and valuable piece of work. And I only have some minor points that should be addressed:

**# FIGURE 1 / IDENTIFICATION OF STUDIES**

 I find the figure to be incomplete.

 In the first pane, the total number of records found is: 105 + 154 + 159 + 35 = 453 (across the three databases + via snowball sampling).

 On the right-hand side, it is then depicted that 6 (or 12?) records were removed as duplicates.

 First, it is unclear what the relevant number is. For example,  the first entry under “duplicate records removed”  reads (n=6 (3 removed)). I find this ambiguous. Does n = 6 refer to the number of duplicate records, or to the number actually removed? Because if 6 duplicates were identified and 3 duplicates were actually removed, that would mean that 3 duplicates were retained?

 Second, the math does not work out, because that would be 453-6 = 447 records left for screening (or 453-12 = 441 – depending on which number is used).  However, the next box states that 213 records were screened, and not 400+.

 This means that 234 records suddenly disappeard, and the flow diagram does not explain what happened to these records.

 I assume that these were removed based on some additional screening/filtering procedure, potentially the DOI matching (as described on p. 5), but this is not clear from the figure.

 Please clarify what happened to these records and adapt the figure accordingly. In particular, add how many duplicates were actually removed.  

**# RESULTS**

 ## EVALUATIVE DIMENSION EXAMPLE

 I wonder about the example the authors cite for the normative category, i.e. the category that captures definitions that view CTs as (socially) harmful / portray them in a negative light / view them as undesirable.

 The cited example is: “Brotherton and French (2014, 238), for instance, use the following definition: ‘an unverified and relatively implausible allegation of conspiracy, claiming that significant events are the result of a secret plot carried out by a preternaturally sinister and powerful group of people’” (p. 8).

 However, in my understanding of the category scheme used by the authors, “unverified and relatively implausible allegation of a conspiracy” would fall under the epistemic dimension, which is described as including definitions where CTs are attributed to have “weak evidence” (p. 8).

 Similarly, “preternaturally sinister ... group of people” seems to describe the actors involved in the conspiracy as malevolent, which should, if I understand the category scheme correctly, fall under the “constitutive features” dimension (and should’ve been  coded as “malevolence”).

 If I am mistaken at some point, please clarify the coding scheme, in particular what is included under which dimension/category. At the moment, I find it somewhat difficult to understand why this particular definition is used as an example of the evaluative dimension.

 Related to that: if a codebook with coding rules exists, it would be great to share this alongside the dataset as part of the replication material.

**## CONSTITUTIVE FEATURES**

 I see some potential to explore variation in more depth. For example whether there us variation over time? Could it be that definitions become more complex over the years, or that certain attributes are considered only in newer definitions?

 A thought that may be potentially interesting for future research - not necessarily in this manuscript, though:

 In the genealogical mapping, the authors write that a substantial proportion of articles draws their definition from a limited number of studies (the central nodes). Could you trace whether all elements of the original definition are retained, or whether subsequent articles drop or add certain elements?

 For example, the authors write: "These patterns indicate that a shared core—intentional, secret coordination by multiple actors—anchors the genealogical centre of the field, while other elements such as power or epistemic quality are variably attached." (p. 10)

 However, these 3 elements seem to be at the heart of the definitions by Keeley (1999), Coady (2003), Bale (2007), Sunstein and Vermeule (2009), and Douglas et al. (2019). As other studies use more elements in their definitions, it looks to me as if these later definitions may have expanded these definitions.

**# GENEALOGICAL MAPPING**

 I also wonder about the points in the network graph that have no connection.

 It would be helpful to clarify what an unconnected node represents in the network graph. Does it mean that the article did not draw on other literature for its definition? Or does it rather means that the article’s definition has no identifiable relationship to another definition in the **sample**? Or does it depend?

 I assume that most of these studies give a definition that also comes from some other study. Could you explain why these studies did not make it into yours sample via the final stage of datacollection through snowball sampling? Did they not meet your inclusion criteria? Or did the just not make it in the mapping effort? Perhaps you could give the n of the maiing effort to show whether all 107 studies were mapped (or not). 

**## ADDITIONAL PLOTS IN SUPPLEMENT**

 The authors write in their abstract that “genealogical mapping shows that frequently reused definitions share these features but differ in their evaluative and epistemic characterisation.” However, this is not visible in the network graph in Figure 2.

 Perhaps the authors did this analysis but did not provide the relevant plots in the article. It would be helpful to color-group the records in the genealogical mapping not by article type (article, review, note), which does not seem to tell us very much, but by category (and dimension), such as neutral vs. normative, etc. Maybe you could provide such additional plots. / analyses as a supplementary material.

Are sufficient details of the methods and analysis provided to allow replication by others?

Partly

Are the rationale for, and objectives of, the Systematic Review clearly stated?

Yes

Is the statistical analysis and its interpretation appropriate?

Yes

Are the conclusions drawn adequately supported by the results presented in the review?

Yes

Reviewer Expertise:

research on CT belief and democracy / survey research

I confirm that I have read this submission and believe that I have an appropriate level of expertise to confirm that it is of an acceptable scientific standard, however I have significant reservations, as outlined above.

# Response

**Published:** 2026-10-06

**Authors:** Turkay Nefes

**Affiliations:** Institute of Public Goods and Policies (IPP), Spanish Scientific Research Council, Madrid, Community of Madrid, Spain

Dear Dr Küppers, Thank you very much for your careful and constructive review. We greatly appreciate your assessment of the article and the dataset, as well as your thoughtful suggestions for improving the clarity of the analysis and its presentation. Please find our responses below.

**Comment 1: Figure 1 / Identification of studies** **Response: **Thank you. You were correct that Figure 1 contained an inconsistency. On rechecking the data, we found that the figures in the first panel had been taken from an earlier version of the database. This did not affect the final corpus of 107 studies. We have corrected Figure 1 and Table 1 using the data. The four sources contained 569 source-level records, corresponding to 213 unique documents after deduplication; 106 were excluded, leaving 107 studies in the review. Thank you for drawing our attention to this.

**Comment 2: Evaluative dimension example** **Response: **Thank you. You are correct that “unverified and relatively implausible” reflects the epistemic dimension and that “sinister” and “powerful” also relate to constitutive features. The five dimensions are analytically distinct but complementary, so the same definition can be characterised across dimensions. In the Brotherton and French definition, the formulation, particularly “sinister”, also contributes to its normative evaluative character. We have clarified this multidimensional coding logic in the Methods. The publicly available dataset reports the coded analytical dimensions for each definition. No separate codebook was used; the coding matrix contained in the dataset records the dimension-level coding for each definition. We have clarified the coding procedure in the article and refer explicitly to the dataset: González-Albo, B., Martínez Escobar, J., & Nefes, T. S. (2025). Bibliographic data from publications with definitions of conspiracy theory and characterisation of the definitions. DIGITAL.CSIC. https://doi.org/10.20350/digitalCSIC/17922.

**Comment 3: Constitutive features** **Response: **Thank you for this helpful suggestion. The article focuses on how conspiracy theories are defined rather than on temporal change. We did not observe a clear temporal pattern in the corpus, although we did not conduct a systematic longitudinal analysis. Your point about the shared core is also well taken. Intentionality, secrecy, and coordination recur in the central definitions, and later studies build on these formulations while sometimes adding further elements. Tracing how such elements are retained, dropped, or added across subsequent studies would be a valuable direction for future research.

**Comment 4: Genealogical mapping** **Response: **Thank you for this helpful point. An unconnected node represents a definition for which no citation relationship with another definition in the analytical corpus was identified. This does not mean that the article did not cite other literature. In some cases, authors formulate their own definition without citing a prior definitional source; in others, cited works do not themselves meet the inclusion criteria for the definitional corpus. All 107 definitions in the analytical corpus were included in the mapping. We have clarified this in the Methods.

**Comment 5: Additional plots in supplementary material** **Response: **Thank you for this suggestion. Because individual definitions combine characteristics across several dimensions, a single colour-coded network would not adequately represent that multidimensional variation. Separate networks for each dimension would also add several figures without necessarily improving interpretation. We have therefore retained the existing presentation of Figure 2, while the evaluative, epistemic, and constitutive differences are addressed in the accompanying text.

 Thank you again for your very helpful comments and for your generous assessment of our work.

 Best regards, Türkay Salim Nefes *on behalf of the authors*

# Reviewer report: Reviewer response for version 1

**DOI:** 10.21956/openreseurope.25235.r74047

**Published:** 2026-06-17

**Authors:** Kenzo Nera

**Affiliations:** 1 Universite Libre de Bruxelles, Brussels, Belgium

It is difficult to emphasize enough how much research sorely needs this contribution, and it is most welcome as conspiracy theory research becomes more self-critical and strives for theoretical integration. I commend the authors for their work and hope it will have an important impact on the field. I have some moderate to minor points that need to be addressed; some of them might require work, but all of them should be highly manageable and do not call into question the core contribution of the article.

 For each dimension of the conspiracy definition (see Table 1), it would be useful to know the relative prevalence of each “subgroup” in absolute values and as a percentage of the definition corpus. Knowing if some approaches are endorsed by the vast majority and others by tiny minorities would be extremely valuable information. For instance, regarding the ontological dimension, I was puzzled to read that some authors define conspiracy theories as “social action” – and I am wondering about the prevalence of such bizarre definitions. Since the word “theory” suggests an intellectual construction, how could a conspiracy *theory *be the plot/conspiracy itself? Note that Wood et al. (2012) define conspiracy theories as a “proposed plot by powerful groups and organizations,” which is ambiguous: the word “proposed” suggests that the plot is embedded in some kind of narrative or belief. The authors may consider discussing this a bit further.

 On page 8, the authors mention examples of how conspiracy theories are epistemically characterized: “Common formulations include references to ‘discounting contradictory evidence’, ‘self-insulating against disconfirmation’, and ‘fallacious logic’.” Providing references for each citation would be highly valuable.

 In the “constitutive features” section, the authors argue that sometimes, the only definitional criterion is opposition to authoritative accounts. As an example, they cite Harris (2022), who defines conspiracy theories as “theories that allege conspiracies and conflict with the claims of relevant epistemic authorities, where epistemic authority is a matter of credentials and positions.” In my view, the concept of “conspiracy” encompasses many of the constitutive features (coalition, secrecy, etc.). Perhaps the authors might consider discussing the notion that some of the features are directly derived from the concept of conspiracy. This would explain why these features are at the core of most definitions. Thus, definitions of conspiracy theories define them by (1) paraphrasing what a conspiracy is, with (2) an optional selection of features (e.g., opposition to authoritative accounts) sprinkled on top.

 The “scope” dimension is ambiguous. At first, I thought it would designate the extent to which definitions make a distinction between conspiracy theories and conspiracy claims in general – the latter being much broader in scope. Yet, the dimension refers to the scale, or topic, of the alleged conspiracies, with some definitions focusing on significant societal events (i.e., in this approach, a conspiracy in the workplace would not count as a conspiracy theory). This distinction makes sense because my initial understanding is redundant with the constitutive features section. The authors may want to tweak the wording of the section to disambiguate it (perhaps using “topic specificity” instead of “scope” – or something along those lines).

 The resolution of the article network (Figure 2) is too low to be readable. I do not know if it is a feature of the platform or an issue with the uploaded figure; if it is the latter, the authors may consider uploading a high-resolution figure.

 On page 9, you mention that “Malevolent intent is not required by Keeley (1999), Coady (2003), or Douglas et al. (2019). Bale’s (2007) contribution differs in kind: rather than offering a definition, he distinguishes between epistemically warranted conspiratorial explanations and what he terms bogus conspiracy theories...” This is correct, but the authors might mention that Keeley’s piece actually proposes to distinguish between conspiracy theories and unwarranted conspiracy theories – an effort similar to Bale’s.

 On page 10, the authors write: “Sunstein and Vermeule (2009) embed negative evaluative and epistemic judgments, portraying conspiracy theories as systematically unreliable and socially harmful.” While these authors are extremely critical of conspiracy theories, their definition itself does not encompass a negative evaluation. That may be a problem because it potentially creates a mismatch between the definition and implicit assumptions (regarding this mismatch, see Nera & Schöpfer, 2023, Theory & Psychology).

 The authors conclude that most studies use a neutral, non-normative definition, which contrasts with the fact that “Institutional definitions—such as those used by the European Commission—often embed assumptions of inherent malevolence and epistemic deficiency.” However, I believe that researchers – and perhaps psychologists in particular – are partly responsible for this contamination of definitions by normative judgments: we mobilize neutral definitions, yet we study almost exclusively conspiracy theories that are problematic (e.g., poorly substantiated, socially dangerous, etc). This creates a mismatch between what we claim to be studying and what we are actually studying. I apologize for the shameless self-promotion, but I believe this review further stresses the need to reflect on the potential mismatch between the definitions we mobilize and the conspiracies we are studying – a point we already discussed in Nera and Schöpfer (2023; see the section “The necessity of a refined approach to defining conspiracy theories in psychology”). I believe this should be mentioned in the closing comments.

 I again applaud the authors for their work.

 Kenzo Nera (signed review)

Are sufficient details of the methods and analysis provided to allow replication by others?

Yes

Are the rationale for, and objectives of, the Systematic Review clearly stated?

Yes

Is the statistical analysis and its interpretation appropriate?

Yes

Are the conclusions drawn adequately supported by the results presented in the review?

Yes

Reviewer Expertise:

Social psychology of conspiracy theories

I confirm that I have read this submission and believe that I have an appropriate level of expertise to confirm that it is of an acceptable scientific standard, however I have significant reservations, as outlined above.

# Response

**Published:** 2026-08-11

**Authors:** Turkay Nefes

**Affiliations:** Institute of Public Goods and Policies (IPP), Spanish Scientific Research Council, Madrid, Community of Madrid, Spain

Dear Dr. Nera, Thank you very much for your constructive and helpful comments. We greatly appreciated the care with which you engaged with the paper and found your suggestions very useful for clarifying both the analysis and its presentation. Please find our responses to each of your comments below.

**Comment 1: Frequencies, percentages and the “social action” category**

 For each dimension of the conspiracy definition (see Table 1), it would be useful to know the relative prevalence of each “subgroup” in absolute values and as a percentage of the definition corpus. Knowing if some approaches are endorsed by the vast majority and others by tiny minorities would be extremely valuable information. For instance, regarding the ontological dimension, I was puzzled to read that some authors define conspiracy theories as “social action” – and I am wondering about the prevalence of such bizarre definitions. Since the word “theory” suggests an intellectual construction, how could a conspiracy theory be the plot/conspiracy itself? Note that Wood et al. (2012) define conspiracy theories as a “proposed plot by powerful groups and organizations,” which is ambiguous: the word “proposed” suggests that the plot is embedded in some kind of narrative or belief. The authors may consider discussing this a bit further.

**Response:** Thank you. We have added frequencies and percentages for the categories (see Table 3). We also re-examined the ontological classifications, focusing on what each definitional formulation identifies a conspiracy theory itself as, rather than on the actions attributed to the alleged conspirators. We agree that Wood et al.’s (2012) formulation, “a proposed plot”, is ambiguous. On reconsideration, we interpret “proposed” as indicating a plot that is posited. We have therefore reclassified this definition as an explanatory/discursive formulation. We also revised the Results (pp. 11-12).

**Comment 2: References for quoted epistemic characterisations**

 On page 8, the authors mention examples of how conspiracy theories are epistemically characterized: “Common formulations include references to ‘discounting contradictory evidence’, ‘self-insulating against disconfirmation’, and ‘fallacious logic’.” Providing references for each citation would be highly valuable.

**Response: **Thank you. We revised this passage (p. 12).

**Comment 3: Features inherited from the concept of conspiracy**

 In the “constitutive features” section, the authors argue that sometimes, the only definitional criterion is opposition to authoritative accounts. As an example, they cite Harris (2022), who defines conspiracy theories as “theories that allege conspiracies and conflict with the claims of relevant epistemic authorities, where epistemic authority is a matter of credentials and positions.” In my view, the concept of “conspiracy” encompasses many of the constitutive features (coalition, secrecy, etc.). Perhaps the authors might consider discussing the notion that some of the features are directly derived from the concept of conspiracy. This would explain why these features are at the core of most definitions. Thus, definitions of conspiracy theories define them by (1) paraphrasing what a conspiracy is, with (2) an optional selection of features (e.g., opposition to authoritative accounts) sprinkled on top.

**Response: **Thank you. We revised this section to clarify that some constitutive features partly reflect their inheritance from the concept of conspiracy (pp. 13-14). In particular, we note that the historical meaning of conspirare foregrounds acting together, which helps explain the prominence of coalition in definitions of conspiracy theory. We also clarify that contradiction of dominant explanations is not implicit in the concept, which may help explain why it sometimes appears independently.

**Comment 4: Ambiguity of the “scope” dimension**

 The “scope” dimension is ambiguous. At first, I thought it would designate the extent to which definitions make a distinction between conspiracy theories and conspiracy claims in general – the latter being much broader in scope. Yet, the dimension refers to the scale, or topic, of the alleged conspiracies, with some definitions focusing on significant societal events (i.e., in this approach, a conspiracy in the workplace would not count as a conspiracy theory). This distinction makes sense because my initial understanding is redundant with the constitutive features section. The authors may want to tweak the wording of the section to disambiguate it (perhaps using “topic specificity” instead of “scope” – or something along those lines).

**Response: **Thank you. We renamed the dimension “Scope of the Explained Phenomenon” and revised the section (pp. 14-15). We have also revised the corresponding description in Table 2.

**Comment 5: Resolution of Figure 2**

 The resolution of the article network (Figure 2) is too low to be readable. I do not know if it is a feature of the platform or an issue with the uploaded figure; if it is the latter, the authors may consider uploading a high-resolution figure. 

**Response: ** Thank you. We replaced Figure 2 with a higher-quality PNG version to improve the readability.

**Comment 6: Keeley and unwarranted conspiracy theories**

 On page 9, you mention that “Malevolent intent is not required by Keeley (1999), Coady (2003), or Douglas et al. (2019). Bale’s (2007) contribution differs in kind: rather than offering a definition, he distinguishes between epistemically warranted conspiratorial explanations and what he terms bogus conspiracy theories...” This is correct, but the authors might mention that Keeley’s piece actually proposes to distinguish between conspiracy theories and unwarranted conspiracy theories – an effort similar to Bale’s.

**Response: **Thank you. We revised this passage to clarify the similarity between Keeley (1999) and Bale (2007) (p. 16).

**Comment 7: Sunstein and Vermeule’s explicit definition**

 On page 10, the authors write: “Sunstein and Vermeule (2009) embed negative evaluative and epistemic judgments, portraying conspiracy theories as systematically unreliable and socially harmful.” While these authors are extremely critical of conspiracy theories, their definition itself does not encompass a negative evaluation. That may be a problem because it potentially creates a mismatch between the definition and implicit assumptions (regarding this mismatch, see Nera & Schöpfer, 2023, Theory & Psychology).

**Response: **Thank you. We revised this passage accordingly (p. 17).

**Comment 8: Mismatch between neutral definitions and selected cases**

 The authors conclude that most studies use a neutral, non-normative definition, which contrasts with the fact that “Institutional definitions—such as those used by the European Commission…” However, I believe that researchers are partly responsible for this contamination of definitions by normative judgments: we mobilize neutral definitions, yet we study almost exclusively conspiracy theories that are problematic (e.g., poorly substantiated, socially dangerous, etc). This creates a mismatch between what we claim to be studying and what we are actually studying. I apologize for the shameless self-promotion, but I believe this review further stresses the need to reflect on the potential mismatch between the definitions we mobilize and the conspiracies we are studying – a point we already discussed in Nera and Schöpfer (2023)...

**​​​​​​​Response: **Thank you. We added this to the Discussion (pp. 19-20). Drawing on Nera and Schöpfer (2023), we now note that broad definitions may be applied to a narrower set of empirical cases.

# Reviewer report: Reviewer response for version 1

**DOI:** 10.21956/openreseurope.25235.r74052

**Published:** 2026-06-01

**Authors:** Nuh Akçakaya

**Affiliations:** 1 Selçuk Üniversitesi, Konya, Turkey

**Peer Review Report**

*How conspiracy theories are defined: A systematic qualitative review of academic definitions* Nefes, Martínez Escobar, and González-Albo (2026)

**Approval Status: Approved**

**Questionnaire Responses**

**Are the rationale for, and objectives of, the Systematic Review clearly stated?** **Yes.** 

 The rationale is clearly articulated in the introduction. The authors identify a real gap — the lack of systematic analysis of how conspiracy theories are defined — and connect it to both academic and policy implications. The two research questions are explicitly stated and consistently addressed throughout the paper.

**Are sufficient details of the methods and analysis provided to allow replication by others?** **Partly.** The search strategy, screening procedure, and coding approach are well documented and the dataset is publicly available. That said, two points need attention. The snowball sampling procedure is not accompanied by any discussion of its limitations, and the methodological basis for the genealogical mapping is underspecified — in particular, how centrality was operationalised is never explained. Both points are discussed further below.

**Is the statistical analysis and its interpretation appropriate?** **Not applicable.** 

 This study uses qualitative content analysis and genealogical mapping. No statistical analysis is involved, and none would be expected here.

**Are the conclusions drawn adequately supported by the results presented in the review?** **Yes.** 

 The five analytical dimensions are applied consistently and the main conclusion — that definitional diversity is reproduced through citation practices — follows naturally from the findings. One suggestion: the paper's central recommendation would carry more weight if the authors discussed the practical trade-offs of different definitional types more explicitly. This is elaborated below.

**Summary**

 This paper looks at how conspiracy theories are defined in peer-reviewed academic literature and maps the conceptual variation across 107 publications. The authors apply qualitative content analysis to identify five recurring dimensions along which definitions differ, and complement this with a genealogical mapping of citation relationships to examine how particular formulations become dominant over time. The paper is clearly written and the findings are well presented. I am happy to recommend it for approval.

**Assessment:**

**Literature and Framing**

 The paper engages seriously with the relevant literature. More importantly, it addresses a problem that goes beyond what standard bibliometric work typically handles. The conspiracy theory field has built up a large and somewhat messy body of definitional work, with competing formulations moving across disciplines without much critical scrutiny. Treating this situation as the object of analysis rather than a background condition is a sensible and overdue move. The framing is honest about what the paper does and does not claim to do.

**Study Design**

 The combination of qualitative content analysis and genealogical mapping works well for the research questions. Using three databases is a reasonable way to reduce disciplinary bias, and the screening procedure is documented clearly enough to follow. Criterion sampling and the focus on explicit, author-endorsed definitions are appropriate choices given the study's aims.

**Results and Conclusions**

 The five dimensions are applied consistently and the results section is well organised. The genealogical analysis adds something that the content analysis alone could not — it shows that conceptual diversity is not just a feature of the field at large but persists even among the most frequently cited formulations. The policy discussion at the end is reasonable, though it could be pushed a little further.

**Specific Comments:**

**Abstract Structure**

 The Results section of the abstract runs together findings from two different methods without signalling which finding comes from which. Qualitative content analysis and genealogical mapping are doing different things, and a reader encountering the abstract for the first time cannot easily tell them apart. Two sentences — one for each method — would sort this out without much effort.

**Methods/Snowball Sampling**

 The paper does not discuss the limitations of snowball sampling. Since this method follows existing citation trails, definitionally relevant but less-cited works may not have been picked up. A sentence acknowledging this would fit naturally in the limitations section, which is otherwise quite candid about what the study cannot claim.

**Genealogical Mapping**

 Figure 2 is interesting and the genealogical approach is a genuine addition to the analysis. The problem is that the basis for identifying central nodes is never explained. The authors point to Keeley (1999), Coady (2003), Bale (2007), Sunstein and Vermeule (2009), and Douglas et al. (2019) as occupying strategic positions, but it is not clear how this was determined. Degree centrality? Betweenness centrality? Something else? A short note on how the network was built and how centrality was measured would make this part of the paper considerably easier to evaluate.

**Evaluative Discussion of Individual Definitions**

 The paper is good at classifying definitions but does not say much about what different definitional choices cost or buy. The recommendation to treat definitions as analytical tools is sensible, but it would be more useful if the authors said something about which tools are suited to which jobs. Neutral-descriptive definitions keep things open but create problems for operationalisation; normative ones draw cleaner lines but risk circular reasoning; minimalist formulations like that of Peels et al. (2023) are arguably circular on their face; broad-scope definitions travel well but lose precision. A short paragraph in the discussion on these trade-offs would give readers something concrete to work with.

**Concluding Remarks**

 The points above are minor and do not affect the paper's main contribution. The study is well designed, clearly presented, and addresses something the field genuinely needs. I hope the comments are useful.

Are sufficient details of the methods and analysis provided to allow replication by others?

Yes

Are the rationale for, and objectives of, the Systematic Review clearly stated?

Yes

Is the statistical analysis and its interpretation appropriate?

Yes

Are the conclusions drawn adequately supported by the results presented in the review?

Yes

Reviewer Expertise:

My area of expertise is conspiracy theories and conspiracy beliefs. I feel that I am able to assess the entire article, including its theoretical framework, qualitative content analysis, genealogical mapping, and conclusions.

I confirm that I have read this submission and believe that I have an appropriate level of expertise to confirm that it is of an acceptable scientific standard.

# Response

**Published:** 2026-08-11

**Authors:** Turkay Nefes

**Affiliations:** Institute of Public Goods and Policies (IPP), Spanish Scientific Research Council, Madrid, Community of Madrid, Spain

Dear Dr. Akçakaya, Thank you very much for your constructive and helpful comments. We greatly appreciated your suggestions, which helped us improve the clarity and presentation of the article. Please find our responses to each of your comments below.

**Comment 1: Abstract structure**

 The Results section of the abstract runs together findings from two different methods without signalling which finding comes from which. Qualitative content analysis and genealogical mapping are doing different things, and a reader encountering the abstract for the first time cannot easily tell them apart. Two sentences — one for each method — would sort this out without much effort.

**Response:** Thank you. We revised the Results section of the abstract to distinguish more explicitly between the findings of the qualitative content analysis and genealogical mapping.

**Comment 2: Limitations of snowball sampling**

 The paper does not discuss the limitations of snowball sampling. Since this method follows existing citation trails, definitionally relevant but less-cited works may not have been picked up. A sentence acknowledging this would fit in the limitations section, which is otherwise quite candid about what the study cannot claim.

**Response: **Thank you. We added a limitation concerning the snowball-sampling procedure. We now clarify that broad searches across three major bibliographic databases constituted the primary search strategy, while snowball sampling was used as a complementary method to identify additional definitions. We also acknowledge that this procedure may underrepresent definitions that are less frequently cited or weakly connected to established citation networks.

**Comment 3: Genealogical mapping and centrality**

 Figure 2 is interesting and the genealogical approach is a genuine addition to the analysis. The problem is that the basis for identifying central nodes is never explained. The authors point to Keeley (1999), Coady (2003), Bale (2007), Sunstein and Vermeule (2009), and Douglas et al. (2019) as occupying strategic positions, but it is not clear how this was determined. Degree centrality? Betweenness centrality? Something else? A short note on how the network was built and how centrality was measured would make this part of the paper considerably easier to evaluate.

**Response: **Thank you. Centrality was measured using in-degree centrality, based on the number of definitions citing each definition (p. 10).

**Comment 4: Trade-offs between definitional approaches**

 The paper is good at classifying definitions but does not say much about what different definitional choices cost or buy. The recommendation to treat definitions as analytical tools is sensible, but it would be more useful if the authors said something about which tools are suited to which jobs. Neutral-descriptive definitions keep things open but create problems for operationalisation; normative ones draw cleaner lines but risk circular reasoning; minimalist formulations like that of Peels et al. (2023) are arguably circular on their face; broad-scope definitions travel well but lose precision. A short paragraph in the discussion on these trade-offs would give readers something concrete to work with.

**Response:** Thank you. We added a paragraph to the Discussion outlining the analytical trade-offs among neutral-descriptive, minimalist, restrictive, broad and narrow definitions (p. 20).
